# Chemogenetic Inhibition of Infralimbic Prefrontal Cortex GABAergic Parvalbumin Interneurons Attenuates the Impact of Chronic Stress in Male Mice

**DOI:** 10.1523/ENEURO.0423-19.2020

**Published:** 2020-10-26

**Authors:** Nawshaba Nawreen, Evelin M. Cotella, Rachel Morano, Parinaz Mahbod, Khushali S. Dalal, Maureen Fitzgerald, Susan Martelle, Benjamin A. Packard, Ana Franco-Villanueva, Rachel D. Moloney, James P. Herman

**Affiliations:** 1 Department of Pharmacology and Systems Physiology, University of Cincinnati, Cincinnati, OH 45237-0506; 2 Neuroscience Graduate Program, University of Cincinnati, Cincinnati, OH 45237-0506; 3 College of Allied Health Sciences, University of Cincinnati, Cincinnati, OH 45237-0506; 4 Veterans Affairs Medical Center, Cincinnati, OH 45221-0506; 5 Department of Neurology and Rehabilitation Medicine, University of Cincinnati, Cincinnati, OH 45237-0506

**Keywords:** DREADDs, GABA, interneurons, parvalbumin, prefrontal cortex, stress coping

## Abstract

Hypofunction of the prefrontal cortex (PFC) contributes to stress-related neuropsychiatric illnesses. Mechanisms leading to prefrontal hypoactivity remain to be determined. Prior evidence suggests that chronic stress leads to an increase in activity of parvalbumin (PV) expressing GABAergic interneurons (INs) in the PFC. The purpose of the study was to determine whether reducing PV IN activity in the Infralimbic (IL) PFC would prevent stress-related phenotypes. We used a chemogenetic approach to inhibit IL PFC PV INs during stress. Mice were first tested in the tail suspension test (TST) to determine the impact of PV IN inhibition on behavioral responses to acute stress. The long-term impact of PV IN inhibition during a modified chronic variable stress (CVS) was tested in the forced swim test (FST). Acute PV IN inhibition reduced active (struggling) and increased passive coping behaviors (immobility) in the TST. In contrast, inhibition of PV INs during CVS increased active and reduced passive coping behaviors in the FST. Moreover, chronic inhibition of PV INs attenuated CVS-induced changes in Fos expression in the prelimbic cortex (PrL), basolateral amygdala (BLA), and ventrolateral periaqueductal gray (vlPAG) and also attenuated adrenal hypertrophy and body weight loss associated with chronic stress. Our results suggest differential roles of PV INs in acute versus chronic stress, indicative of distinct biological mechanisms underlying acute versus chronic stress responses. Our results also indicate a role for PV INs in driving chronic stress adaptation and support literature evidence suggesting cortical GABAergic INs as a therapeutic target in stress-related illnesses.

## Significance Statement

Stress-related diseases are associated with prefrontal hypoactivity, the mechanism of which is currently not known. In this study we showed that by inhibiting prefrontal GABAergic parvalbumin interneurons (PV INs), we can attenuate some of the chronic stress-related phenotypes. Additionally, we showed that modulation of PV IN activity during acute stress had opposing effects on stress coping strategies, suggesting different plasticity mechanisms in PV INs following acute versus chronic stress. Our findings indicate that GABAergic PV INs may be involved in driving stress-related phenotypes and may therefore be an important target for treatment of stress-related illnesses. Our data suggest that reducing PV IN activity to promote prefrontal output may be a potential treatment strategy for stress-related disorders.

## Introduction

Mood disorders [e.g., posttraumatic stress disorder (PTSD) and major depressive disorder (MDD)] are associated with alterations in ventromedial prefrontal cortex (PFC; Broadman area 25) structure, activity and connectivity (Rogers et al., 2004; Drevets et al., 2008; Hasler et al., 2008; Murray et al., 2011; Holmes et al., 2018). To date, no universally efficacious therapeutic strategy exists for these neuropsychiatric conditions, despite having a lifetime prevalence of over 20% (Kessler et al., 2005; Duman and Duman, 2015). Studies in both humans and animal models have shown that chronic stress impairs functioning of the PFC, potentially making prefrontal hypofunction an important factor in the etiology of mood disorders (Drevets et al., 1997; Radley et al., 2006; Duman and Duman, 2015; Li et al., 2011; McKlveen et al., 2016).

Various clinical and preclinical studies implicate altered GABAergic circuitry and prefrontal hypofunction in the generation of depression in humans as well as depression-related behaviors in rodent chronic stress models (Luscher et al., 2011; Duman, 2014; Veeraiah et al., 2014; Musazzi et al., 2015; McKlveen et al., 2016). Recent functional and electrophysiological studies indicate increased infralimbic (IL) PFC (rodent homolog of the human ventromedial PFC) GABAergic transmission (e.g., increased inhibitory synaptic drive and increased expression of GABAergic marker) following chronic variable stress (CVS). These findings suggest that enhanced interneuron (IN) activity may be involved in disruption of prefrontal cortical signaling, leading to over inhibition and prefrontal hypofunction (McKlveen et al., 2016; Shepard et al., 2016; Page et al., 2019).

GABAergic parvalbumin INs (PV INs) synapse onto cell bodies of PFC pyramidal neurons and exert strong control over medial PFC (mPFC) output, maintaining appropriate excitatory/inhibitory (E/I) balance (Cardin et al., 2009; Courtin et al., 2014a; Tremblay et al., 2016) and coordinating oscillatory activity (γ oscillation) required for efficient PFC signaling. Consequently PV INs are well positioned to play an important role in stress-mediated prefrontal dysfunction (Rymar and Sadikot, 2007; Sherwood et al., 2007; DeFelipe et al., 2013; Ferguson and Gao, 2018). Increases in expression and activity of PV INs and enhancement of glutamatergic transmission onto PV INs following chronic stress are associated with prefrontal hypofunction, anxiogenesis, and impaired coping behaviors in forced swim test (FST) (Shepard et al., 2016; Page et al., 2018, 2019; Shepard and Coutellier, 2018). Reduction in PV expression in PFC is also associated with antidepressant efficacy (Ohira et al., 2013; Zhou et al., 2015; Page and Coutellier, 2019). In contrast to chronic stress, acute inhibition of PV INs in the PFC has opposing effects, resulting in increase in passive coping behavior such as learned helplessness in mice (Perova et al., 2015). This suggests differential role of PV INs in response to acute versus chronic stress, indicative of distinct brain circuitry being involved in modulating acute versus chronic stress-mediated phenotypes.

This study was designed to specifically investigate the role of IL PV INs in driving somatic and behavioral manifestations of acute and chronic stress-related phenotypes. We employed a chemogenetic strategy using designer receptors exclusively activated by designer drugs (DREADDs) to specifically inhibit PV INs in the IL mPFC during exposure to acute stress and throughout exposure to a modified CVS paradigm. Our results indicate that acute inhibition of PV INs increases passive and decreases active coping behavior in tail suspension test (TST). In contrast, chronic inhibition of IL PV INs reduces passive and increases active coping behavior in FST. PV IN inhibition attenuates CVS-mediated reduction in Fos expression in stress-related brain regions downstream of the IL. Additionally, we show that inhibition of PV INs prevents CVS-induced somatic effects such as adrenal hypertrophy and body weight loss. These data suggest that IL PV INs play a role in driving behavioral, neuronal and physiological adaptations associated with chronic stress and also indicate differential role of these INs in the context of acute versus chronic stress-related phenotypes.

## Materials and Methods

### Mice

Male breeders from B6 PV-Cre knock-in homozygous mice line (B6;129P2-Pvalbtm1(cre)Arbr/J, JAX stock #017320, The Jackson Laboratory) were bred with WT C57BL/6J females (JAX stock #000664) to generate an in-house colony of heterozygous PV-Cre C57BL/6J at the University of Cincinnati animal housing facility. The PV-Cre mouse line has been characterized extensively in prior publications (Zhao et al., 2019; Cummings and Clem, 2020; Groisman et al., 2020). Mice were maintained under standard conditions (12/12 h light/dark cycle, 22 ± 1°C, food and water *ad libitum*; four mice per cage on arrival) in accordance with the University of Cincinnati Institutional Animal Care and Use Committee, which specifically approved all acute and chronic stress regimens employed in this proposal. Mice were single housed following surgeries and continued to be housed singly throughout the duration of the experiment, to prevent aggression and injury to animals following surgery and during the stress paradigms (Lidster et al., 2019). Enrichment for housing cages included mouse hut and nestlets. All protocols conformed to the Society’s Policies on the Use of Animals in Neuroscience Research. All experiments were performed on adult male mice (∼7.5 months of age at surgery).

### Stereotaxic viral vector injection with AAV vectors

**Figure 1.**
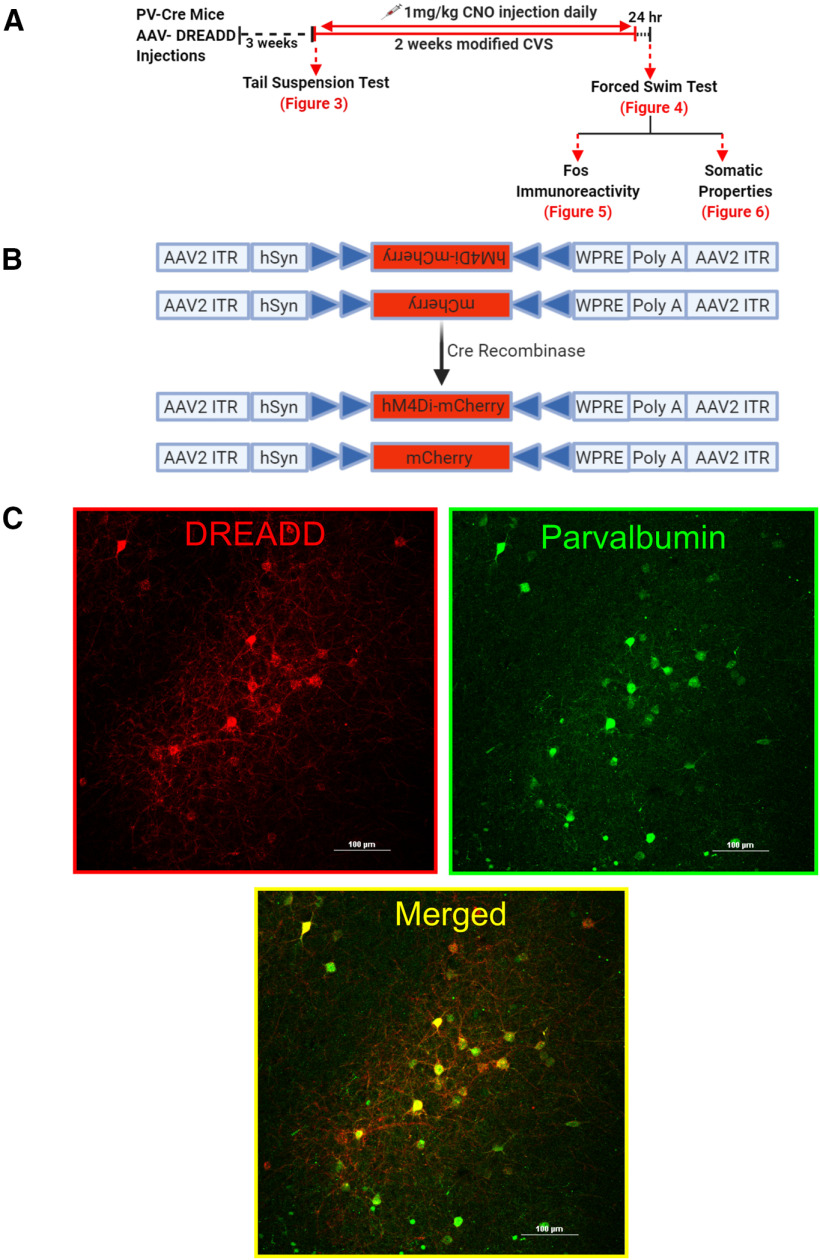
Experimental design and targeting of PV INs in the PFC using DREADDs. ***A***, Experimental design and timeline. C57BL/6J PV-Cre mice ∼7.5 months of age underwent surgery to inject AAV2-hM4Di-mCherry (inhibitory DREADD) or AAV2-mCherry (control virus). Mice were allowed three weeks to recover to enable sufficient time for DREADD expression. Animals were then subjected to CVS procedure twice a day for 14 d or served as controls. The first stressor was a TST to determine acute effects of PV IN inhibition in animals within the CVS group. Animals were dosed with 1 mg/kg CNO before each stressor to inhibit PV INs during the CVS procedure; 24 h after the end of CVS, animals were subjected to FST, following which mice were euthanized, body was perfused, and brains and organs were collected. No CNO was administered during the testing phase in FST. ***B***, Design of AAV2-hSyn-hM4Di-mCherry (top) and AAV2-hSyn-mCherry (bottom) vectors employing the DIO strategy. Two pairs of heterotypic, antiparallel loxP recombination sites (blue triangles) achieve Cre-mediated transgenes inversion and expression under the control of hSyn promoter. ITR, left-inverted terminal repeat; hSyn, human synapsin; WPRE, woodchuck hepatitis DREADD posttranscriptional regulatory element. ***C***, Successful Cre-mediated recombination of DREADDs demonstrated by presence of red mCherry (left); green fluorescence identifies PV INs (right); hM4Di receptors selectively expressed in PV INs as illustrated by mCherry (red) and PV (green) co-expression (merged, yellow, bottom middle image). Scale bar: 100 μm.

PV-Cre mice were anaesthetized with isoflurane, scalp shaved and placed in the stereotaxic frame. The incision site was disinfected using chlorohexidine and 70% ethanol. An incision at the midline was made using a single-edged blade. Cre-dependent adeno-associated virus 2 (AAV2) vectors AAV2-hsyn-DIO-hM4D(Gi)-mCherry (Gift from Bryan Roth; Addgene viral prep #44362-AAV2) and AAV2-hsyn-DIO-mcherry (Gift from Bryan Roth; Addgene viral prep #50459-AAV2) were injected bilaterally at a volume of 300 nl (∼10^12^ genome copies/ml) into the IL mPFC. A pilot study was conducted (data not shown) to optimize viral load, volume and stereotactic coordinates to ensure viral spread is restricted to the IL. We specifically chose AAV serotype 2, which has a limited volume of spread enabling us to primarily target the IL and prevent spread to other regions (Burger et al., 2004; Cearley and Wolfe, 2006). The coordinates used were as follows: (anterior/posterior range defined as +1.78 mm anterior to bregma, medial–lateral range defined as ±0.2 mm lateral to the midsagittal suture; dorsal–ventral range defined as −2.9 mm ventral to skull (Franklin and Paxinos, 2007). Viruses were infused using a 2-μl Hamilton syringe at a rate of 60 nl/min for 5 min. To ensure that virus injection was restricted to bilateral spread in the IL, we took particular care at a few key points: (1) mouse skulls were always carefully vertically leveled based on bregma and λ to ensure bilateral spread, (2) Hamilton syringes were always checked to run properly before injections and were carefully cleaned to remove excess virus particles after viral loading to prevent spread when the needle is lowered into the brain, (3) following infusion, the injector was kept at the site for 8 min to allow for the virus to diffuse and prevent spread to other regions, and (4) after injection, the needle was very slowly removed from the injection site to avoid spreading of the virus throughout the injector. The injection site on the skull was covered with gel foam and incision site sutured; 2.5 mg/kg meloxicam was administered for 3 d following surgery. Behavioral studies and stress protocols were initiated three weeks post injection to allow sufficient time for viral expression. Diagrammatic representation of experimental timeline is outlined in Figure 1*A*.

### Modified CVS procedure

During the CVS procedure, mice were subjected to a series of randomly alternating stressors administered twice daily over a period of 14 d. The CVS procedure used in this study is a modification of prior published protocols from the literature (Ostrander et al., 2009; Ghosal et al., 2017; Wohleb et al., 2018; Cotella et al., 2019), such that each individual stress session did not last for >2 h to ensure that CNO was on-board throughout the stressor (Jendryka et al., 2019). CNO has been shown to reach maximal plasma levels 30 min after intraperitoneal injection and has been shown to rapidly clear from plasma in rodents (Baldessarini et al., 1993; Guettier et al., 2009; MacLaren et al., 2016; Jendryka et al., 2019). As a result, extended stress periods and overnight stressors typically used in CVS paradigms were excluded from the modified CVS paradigm. Additionally, swim stress was not included in the CVS paradigm since animals were tested in FST (a novel stressor) following completion of the CVS procedure. The unpredictable stressors used were as follows: restraint (30 min), cold room exposure (15 min, 4°C), shaker stress (1 h, 100 rpm), hypoxia (30 min, 8% oxygen and 92% nitrogen), Y maze (8 min), shallow water (30 min), wet bedding (2 h), and cage tilt (2 h, 45°). Body weights were measured on days 1, 4, 8, and 14 during the CVS procedure.

### Drug administration

Clozapine N-oxide (CNO; NIMH Chemical synthesis and Drug Supply Program) was used as the DREADD actuator to activate the inhibitory DREADD. CNO was dissolved in 5% dimethyl sulfoxide (Sigma) and then diluted with 0.9% saline and administered intraperitoneally at a dose of 1 mg/kg twice a day, 30 min before start of each stressor. We chose intraperitoneal injections for CNO delivery to effectively control the dose and timing of DREADDs during our stress paradigm. All animals received chronic injection of CNO for 14 d. Previous work has shown that chronic CNO for 14 d does not lead to desensitization of hM4Di DREADDs, confirming efficacy of chronic activation of DREADDs (Soumier and Sibille, 2014; Xia et al., 2017; Stedehouder et al., 2018; Phillips et al., 2019). A maximum time period of 6 h was given between stressors, to allow sufficient time for CNO to be cleared from the body (MacLaren et al., 2016; Jendryka et al., 2019). To ensure inhibiting PV INs had no effects on locomotor activity, total distance traveled and velocity of mice was measured during Y maze task on day 12th of the CVS regimen.

### Behavioral assessments

#### TST

The TST (Can et al., 2011) was used as the first stressor in the CVS group to observe acute effects of inhibiting PV INs on passive coping behavior. Mice were suspended 55 cm above ground using a 17 cm long tape that was attached to a suspension bar, for a total time period of 6 min. Sessions were video recorded from the side to allow full body visualization of mice behaviors-active coping (struggling) behavior, which comprised of strong shaking of the body and movement of all four limbs, and passive coping (immobility) behavior which comprised of not making any active limb movements. Latency to reach immobility was also measured. Behaviors were quantified by an experimenter blinded to the group assignments using behavioral scoring software Kinoscope 3.0.4. Behaviors during the 6-min block were reported.

#### FST

FST was conducted 24 h following completion of the CVS procedure. Mice were placed in a clear cylinder (2-l glass beaker) filled with water (24 ± 1°C, 18-cm depth) for a period of 10 min. Sessions were video recorded from the side to allow full body visualization for total immobility duration, which comprised of not making any active movements or floating in the water without struggling, and total swimming duration, which comprised of moving limbs in an active manner and making circular movements around the cylinder. Behaviors were quantified by an experimenter blinded to the group assignments using behavioral scoring software Kinoscope 3.0.4. Behaviors during the 10-min block were reported. It should be noted that all mice in the study (including CVS and controls) underwent FST as a behavioral test. This includes the mice that had TST in the beginning of the CVS paradigm. To control for a potential effect of chronic exposure to CNO, a separate group of PV-Cre mice were chronically injected with either saline or CNO for 14 d during CVS (2 × 2 design) and then behaviorally tested in the FST.

### Euthanasia and tissue collection

Mice were euthanized with an overdose of sodium pentobarbital after FST, and transcardially perfused with 0.9% saline followed by 4% paraformaldehyde in 0.01 m PBS, pH 7.4. Brains were removed and postfixed in 4% paraformaldehyde at 4°C for 24 h, then transferred to 30% sucrose in 0.01 m PBS at 4°C until processed. Thymi and adrenal glands were collected, cleaned, and weighed from all animals.

### Immunohistochemistry

Brains were sectioned into 30-μm coronal sections using a freezing microtome (−20°C). Sections were collected into 12 wells (1/12) containing cryoprotectant solution [30% sucrose, 1% polyvinyl-pyrolidone (PVP-40), and 30% ethylene glycol, in 0.01 m PBS]. Immunohistochemistry was performed at room temperature (RT) and 0.01 m PBS was used to rinse brain slices before each treatment described below.

#### PV and DREADD co-localization

Targeting of IL mPFC PV neurons and recombination of hM4Di DREADD was verified by co-localization of PV immunoreactivity with virally expressed mCherry fluorescence. Free floating sections were incubated in blocking solution [4% normal goat serum (NGS), 0.1% Triton X-100, 0.1% bovine serum albumin (BSA) in 0.01 m PBS] for 1 h at RT. After that, sections were incubated with rabbit anti-PV (1:1000, Abcam, ab11427) overnight, followed by visualization with donkey- anti-rabbit Alexa Fluor 488 conjugate (1:500, Invitrogen, A11034). Images were acquired using Nikon Confocal Microscope at 40× magnification.

#### Injection site and viral spread

To determine whether virus spread was restricted to the IL, sections were incubated with a rabbit anti-mCherry (1:500, Abcam, ab167453) for 2 h, followed by visualization with goat anti-rabbit Cy5 conjugate (1:500, Invitrogen, A10523). Images were acquired using Carl Zeiss Imager Z1 at 2.5× and 5× magnification. Viral spread was mapped onto its respective focal plane and bregma level by outlining the spread of the infection from confocal images onto corresponding brain atlas illustrations (Lein et al., 2007). Fluorescent cells were counted using semi-automated analysis macro in the ImageJ software package (National Institutes of Health). The threshold was adjusted to detect the fluorescence and cells counted using the Analyze Particle feature. The percentage of infected cells in IL was calculated by dividing the number of mCherry-positive cells in IL by the total number of mCherry-positive cells in a specific focal plane. Brain bregma coordinates used for IL cytoarchitecture were defined in the Franklin and Paxinos mouse brain atlas (third edition; Franklin and Paxinos, 2007).

##### Fos immunoreactivity

Neuronal activation was measured using Fos as a marker. Free floating sections were incubated in 1% sodium borohydride for 20 min and then in 3% hydrogen peroxide in PBS for 20 min. After that, slices were incubated in blocking solution (NGS, 0.3% Triton X-100, 0.2% BSA in 0.01 m PBS) for 1 h. Sections were then incubated with Fos rabbit polyclonal antibody (1:200, Santa Cruz, sc-52) in blocking solution overnight and was followed by incubation in secondary antibody (biotinylated goat anti-rabbit, (1:400; Vector Laboratories, BA1000) in blocking solution for 1 h the next day. Sections were then treated with avidin-biotin horseradish peroxidase complex (1:800 in 0.01 m PBS; Vector Laboratories, PK6100) for 1 h and then developed with an 8-min incubation in 3,3′-diaminobenzidine (DAB)-Nickel solution: 10 mg DAB tablet (Sigma, DF905), 0.5 ml of a 2% aqueous nickel sulfate solution, 20 μl of 30% hydrogen peroxide in 50 ml of 0.01 m PBS. Sections were mounted on superfrost slides (Fisherbrand, Fisher), allowed to dry, dehydrated with xylene, and then coverslipped with DPX mounting medium (Sigma).

Images were acquired using microscope Carl Zeiss Imager Z1 at a 5× objective. For analysis, we counted minimum of three bilateral sections per brain region/animal covering the prelimbic cortex (PrL; bregma 2.80–1.98 mm), basolateral amygdala (BLA; bregma −1.06 to −1.58), and ventrolateral periaqueductal gray (vlPAG; bregma −4.16 to −4.36) as defined in the Franklin and Paxinos mouse brain atlas (Franklin and Paxinos, 2007). The number of Fos-positive nuclei was counted using a semi-automated analysis macro in the ImageJ software package (National Institutes of Health). The macro was generated using the Analyze Particle tool, with a defined common level of background intensity, nuclei circularity and size (previously validated manually). The relative density of the population of immunopositive cells was calculated by dividing the number of Fos-positive cells by the respective brain area.

### Statistical analysis

The experiment was setup as a 2 × 2 study design, with stress (CVS or No CVS) and DREADD (hM4Di or control) as factors with a sample size of *n* = 10 per group (for experimental design and timeline, see Fig. 1*A*). Statistical analyses for FST and Fos protein quantification were performed using a two-way ANOVA with stress (No CVS, CVS) and DREADD (Control, hM4Di) as main factors. TST data were analyzed using Student’s *t* test. FST measurements over time were done using two-way repeated measure ANOVA with stress (No CVS, CVS) and DREADD (Control, hM4Di) as main factors analyzed over time. Tukey’s *post hoc* test was performed in cases with significant interaction between factors. Because specific hypotheses were formed a priori on the effects of CVS within groups, planned comparisons using Fisher’s least significant difference (LSD) were performed in cases with no significant interaction effect. Data were analyzed by STATISTICA 7.0 (Statsoft) and GraphPad Prism 8.1.2 (GraphPad Software). Outliers were detected using the Grubbs’ test (GraphPad Software) and removed from analysis. After exclusion of outliers, data were assessed for normal distribution (Shapiro–Wilk) and appropriate parametric and/or non-parametric tests used. Data are presented as mean ± SEM with statistical significance set at *p *≤* *0.05. See Table 1 for details regarding data structure and type of test used. Superscript letters listed with *p* values correspond to the statistical tests shown in Table 1.

**Table 1:** Data structure, type of test to analyze the data, and observed power of key results

	Data structure	Type of test	Power
a	Normal distribution	Unpaired sample *t* test (struggling)	Main effect hM4Di DREADD : Cohen’s *d* = 1.2
b	Normal distribution	Unpaired sample *t* test (immobility)	Main effect hM4Di DREADD: Cohen’s *d* = 1.3
c	Normal distribution	Unpaired sample *t* test (latency to immobility)	Main effect hM4Di DREADD: Cohen’s *d* = 1.3
d	Normal distribution	Two-way ANOVA (swimming duration)	Main effect of stress: 0.91 Main effect of hM4Di DREADD: 0.55
e	Normal distribution	Two-way repeated measures ANOVA (swimming duration over time)	Main effect stress: Power = 0.91Main effect hM4Di DREADD: Power = 0.55 Main effect of time: Power = 1
f	Normal distribution	Two-way ANOVA (immobility duration)	Main effect stress: Power = 0.85
g	Normal distribution	Two-way repeated measures ANOVA (immobility duration over time)	Main effect stress: Power = 0.85 Main effect time: Power = 1
h	Normal distribution	Two-way ANOVA (PrL)	Main effect stress: Power = 0.98 Interaction (stress × hM4Di DREADD): Power = 0.76
i	Normal distribution	Two-way ANOVA (IL)	Main effect stress: Power = 0.99
j	Normal distribution	Two-way ANOVA (BLA)	Main effect stress: Power = 0.89
k	Normal distribution	Two-way ANOVA (vlPAG)	Main effect stress: Power = 0.94
l	Normal distribution	Two-way ANOVA (adrenal weight)	Main effect stress: Power = 0.90 Interaction (stress × hM4Di DREADD): Power = 0.57
m	Normal distribution	Two-way ANOVA (thymus weight)	Main effect stress: Power = 1
n	Normal distribution	Two-way ANOVA (final body weight)	Main effect stress: Power = 0.70
0	Normal distribution	Two-way repeated measures ANOVA (body weight over time)	Main effect stress: Power = 0.6 Main effect time: Power = 0.9
p	Normal distribution	Unpaired sample *t* test (viral spread in IL)	Main effect hM4Di DREADD in IL: Cohen’s *d* = 1.2

Table depicts the data structure, type of statistical test used, and power of key results for each of the statistical tests used in the manuscript. Each analysis includes a letter indicator linking the test in the table to the analysis in the text.

## Results

### Selective targeting of PV INs achieved using DREADDs

The AAV constructs used in this experiment are shown in Figure 1*B*. Following Cre recombination, the viral construct expresses inhibitory DREADD sequence hM4Di along with a fluorescent reporter (mCherry), allowing visualization of cells undergoing recombination. Control virus was a Cre-inducible mCherry lacking the DREADD hM4Di construct. Cre-mediated recombination of hM4Di-mCherry and cell type specificity were conferred by immunostaining. Expression of red hM4Di-mCherry demonstrates successful recombination (Fig. 1*C*, left). hM4Di-mCherry expression was restricted to PV INs only (Fig. 1*C*, right), confirmed by colocalization of red mCherry with green (Alexa Fluor 488) PV immunostaining, resulting in yellow cells in the merged image (Fig. 1*C*, bottom).

### Targeting of IL and viral spread

**Figure 2.**
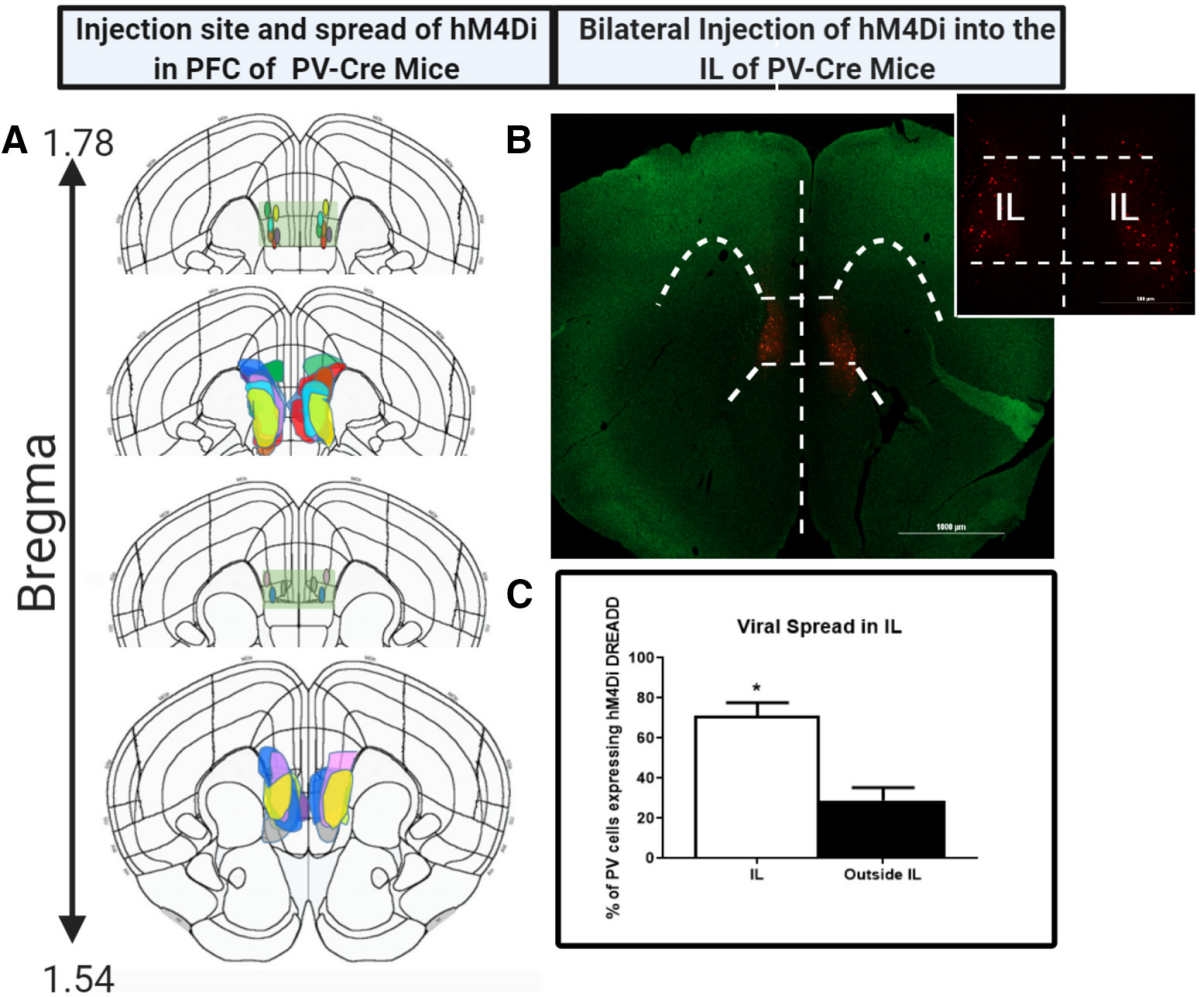
Injection site, viral spread. and targeting of IL. ***A***, Injection site and viral spread mapped onto respective mouse bregma coordinates, following bilateral injection of hM4Di DREADD into the IL of PV-Cre mice (*n* = 9). Light green rectangular box depicts the IL region with the injection sites restricted to the region. Each animal’s injection site and viral spread has been represented by a unique color. ***B***, Representative image from one animal demonstrating the viral spread in PFC detected by red mCherry fluorescence. Image shows the spread was restricted to the IL with the white dashed lines outlining the IL cortex region of the PFC. Scale bars: 1000 and 500 μm. ***C***, Percentage of DREADD transfected PV INs in the IL of PV-Cre mice. The percentage of infected cells in IL was calculated by dividing the number of mCherry-positive cells in IL by the total number of mCherry-positive cells in a specific focal plane and bregma coordinate. Brain bregma coordinates used for IL cytoarchitecture were taken from Franklin and Paxinos (2007). *indicates significant effect *p* < 0.05 compared to outside IL group. Values represent mean ± SEM, *n* = 9 per group.

Figure 2*A* depicts viral injection site and spread of virus determined by the presence of mCherry and far-red (Cy5) fluorescence in animals injected with hM4Di DREADD (*n* = 9). It shows the stereotaxic injection sites and that AAV DREADD expression was primarily restricted to the IL, with some minor spread into the PrL and dorsal peduncular (DP) cortex located above and below IL, respectively. Injection sites and viral spread were located primarily within the bregma range 1.78–1.54 (Franklin and Paxinos, 2007). Figure 2*B* is a representative image from an animal demonstrating red mCherry fluorescence primarily in the IL delineated by white dashed lines. Quantification of percentage of transfected neurons from animals injected with hM4Di DREADD, revealed expression was significantly contained, with ∼72% of transfected cells localized in the IL (*t* = 4.6, df = 16, *p* = 0.0003^p^; Fig. 2*C*).

### Acute inhibition of PV INs: TST

**Figure 3.**
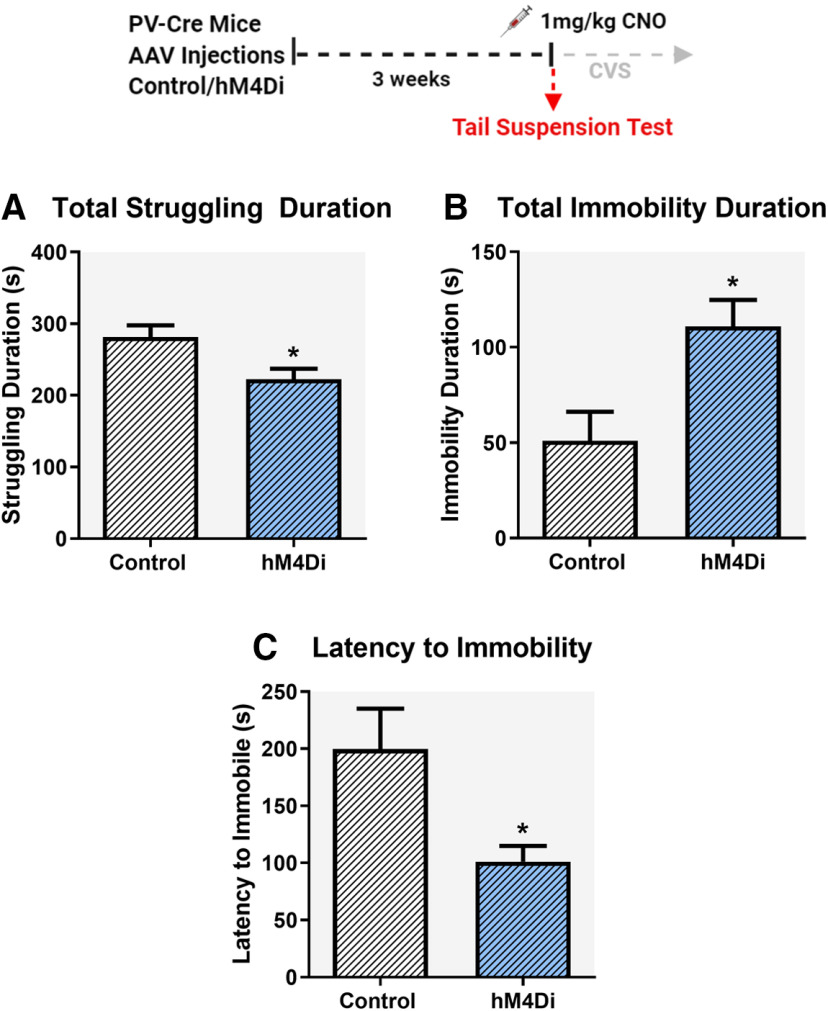
Impact of acute chemogenetic inhibition of PV IN in the IL mPFC on coping behavior in the TST. Acute inhibition of PV INs in the IL mPFC during TST, reduced total time spent struggling (***A***), increased total time spent immobile (***B***), and reduced latency to immobility (***C***). All mice were treated with CNO (1 mg/kg, i.p.) 30 min before TST. Behaviors were analyzed for a total time of 6 min. Values represent mean ± SEM, *n* = 9–10 per group; * indicates significant effect *p* < 0.05 versus corresponding control group.

The behavioral consequences of acute inhibition of PV INs in the IL were tested by using the TST as the first stressor in the CVS paradigm (Fig. 3). Animals were dosed with 1 mg/kg CNO 30 min before the start of TST. We observed significant changes in coping behavior following acute inhibition of PV INs in the IL. Compared with control mice, mice expressing hM4Di showed significant reduction in struggling duration (*t* = 2.7, df = 18, *p* = 0.02^a^; Fig. 3*A*), significant increase in immobility duration (*t* = 2.9, df = 18, *p* = 0.009^b^; Fig. 3*B*) and decreased latency to immobility (*t* = 2.5, df = 17, *p* = 0.02^c^; Fig. 3*C*), respectively.

### Chronic inhibition of PV INs during CVS: Impact on FST

**Figure 4.**
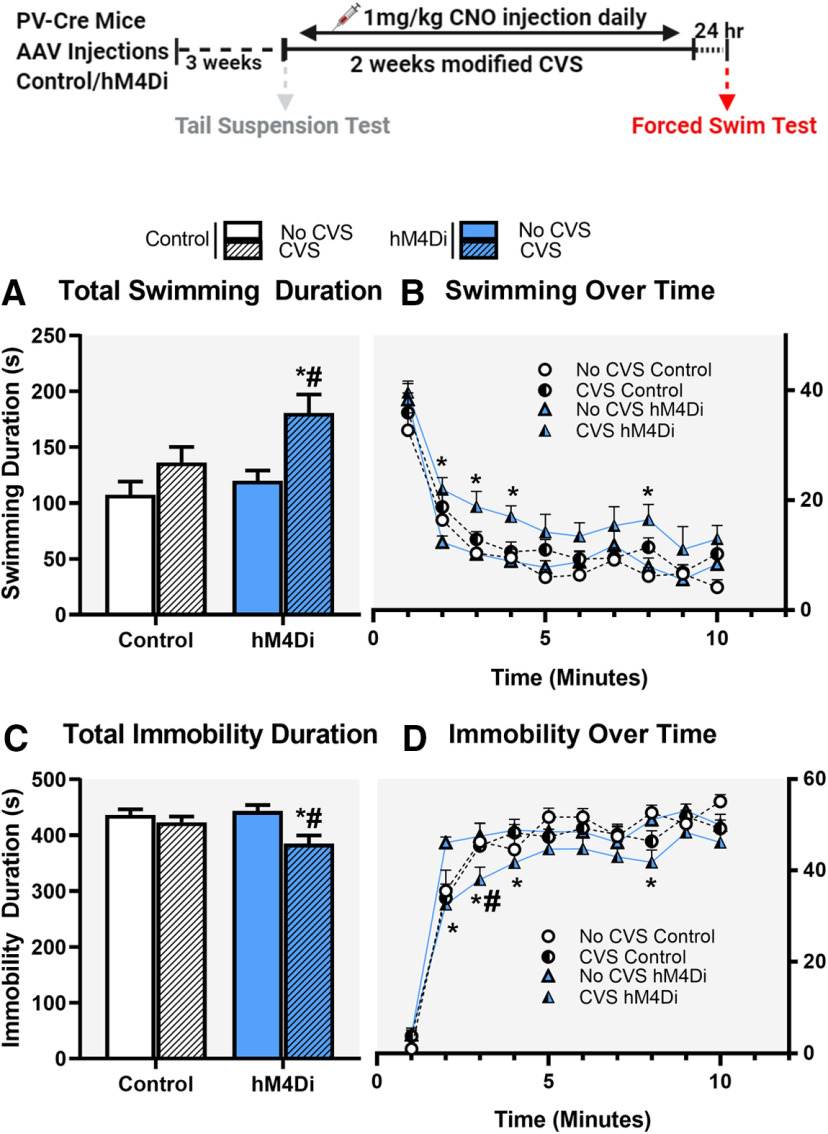
Effects of chronic chemogenetic inhibition of PV INs during CVS on coping behavior in the FST following CVS. Chronic inhibition of PV INs in the IL mPFC during CVS, resulted in increased total time spent swimming (***A***) and decreased total time spent immobile (***C***) in the FST. ***B***, ***D***, Changes in swimming and immobility behavior, respectively, over the 10 min of FST. Values represent mean ± SEM, *n* = 9–10 per group; * indicates planned comparison significant effect *p* < 0.05 versus corresponding No CVS hM4Di group; # indicates planned comparison significant effect *p* < 0.05 versus corresponding CVS Control group. Extended Data Figure 4-1 demonstrates chronic inhibition of PV INs did not affect locomotor activity. Extended Data Figure 4-2 demonstrates chronic CNO administration did not lead to any changes in FST behavior following CVS or control. Extended Data Figure 4-3 demonstrates CVS effects on FST did not depend on the age of the animal and also shows chronic injection stress and housing conditions had no effect on FST behavior in control animals.

Animals were tested for coping behaviors in the FST 24 h after cessation of CVS. Our purpose was to test whether inhibition of PV INs during the chronic stress regimen could block the aggregate effect of repeated stress on subsequent coping behavior (FST used as a novel stressor), brain activation patterns (Fos expression) and somatic endpoints (organ and body weights). All subjects received viral (Control or hM4Di) and CNO treatments, and because CNO was only administered during CVS, any phenotypes observed during FST were interpreted as reflecting an impact of PV IN manipulation during CVS on subsequent stress coping behavior. We observed significant differences in FST coping behaviors following chronic inhibition of PV INs during CVS. Specifically, chronic PV IN inhibition during stress increased active coping (swimming) and reduced passive coping (immobility) behaviors in the FST. Two-way ANOVA of total swimming duration showed a significant main effect of stress (*F*_(1,35)_ = 11.7; *p* = 0.002^d^; Fig. 4*A*) and DREADD (*F*_(1,35)_ = 4.7; *p* = 0.037^d^) but no stress × DREADD interaction (*F*_(1,35)_ = 1.47; *p* = 0.2). Planned comparisons revealed a significant increase in swimming duration in the CVS hM4Di group compared with both CVS Control (*p* = 0.02; Fig. 4*A*) and No CVS hM4Di group (*p* = 0.002; Fig. 4*A*). Analysis of swimming behavior over time showed a main effect of stress (*F*_(1,35)_ = 11.7; *p* = 0.002^e^; Fig. 4*B*), DREADD (*F*_(1,35)_ = 4.7; *p* = 0.037^e^) and time (*F*_(9,315)_ = 68.2; *p* < 0.0001^e^) but no interaction effects were observed among the three groups time × stress × DREADD (*F*_(9,315)_ = 0.6; *p* = 0.78). Planned comparisons revealed significant increase in swimming duration in the CVS hM4Di group at 2-, 3-, 4-, and 8-min time points compared with No CVS hM4Di group (*p* = 0.004, *p* = 0.009, *p* = 0.01, and *p* = 0.009, respectively; Fig. 4*B*).

10.1523/ENEURO.0423-19.2020.f4-1 Extended Data Figure 4-1 Effect of chronic inhibition of PV IN on locomotor activity. Chronic inhibition of PV INs had no effect on locomotor activity as demonstrated by no change in distance travelled (***A***) or velocity (***B***) in hM4Di group compared with control group in a Y maze task. Values represent mean ± SEM, *n* = 9–10 per group (*p* > 0.05). Download Figure 4-1, TIF file.

10.1523/ENEURO.0423-19.2020.f4-2 Extended Data Figure 4-2 Effect of chronic dosing of CNO and saline in control and CVS animals in FST. Chronic CNO administration had no effect on immobility (***A***) or swimming duration (***B***) in FST and also did not have any effect on body weight (***C***) in either CVS or Control groups. Values represent mean ± SEM; *n* = 8 per group (*p* > 0.05); * indicate planned comparisons significant effect *p* < 0.05 versus corresponding No CVS Control groups. Download Figure 4-2, TIF file.

10.1523/ENEURO.0423-19.2020.f4-3 Extended Data Figure 4-3 Effect of age, chronic injection, and housing condition in FST. ***A***, ***B***, Effect of age on FST immobility duration. Analysis of z scores showed that CVS had no effect on FST behavior in different age groups (*t* = 0.4, df = 16, *p* = 0.7; ***B***). Younger and older animals were run in separate experiments. Z score calculation was done as described previously using formula z = (X-μ)/σ to indicate how many SDs (σ) each CVS immobility duration value (X) was from the mean of control group (μ) for each age (Guilloux et al., 2011). ***C***, ***D***, Effect of chronic injection stress and housing conditions on immobility duration in FST in control animals. There was no significant effect of chronic injection stress (*t* = 0.8, df = 14, *p* = 0.4; ***C***) or housing conditions (*t* = 0.2, df = 14, *p* = 0.9; ***D***) in FST behavior. Values represent mean ± SEM; *n* = 8–10 per group; * indicates significant effect *p* < 0.05 versus corresponding control group. Download Figure 4-3, TIF file.

Two-way ANOVA of total immobility duration showed a significant main effect of stress (*F*_(1,35)_ = 9.5; *p* = 0.004^f^; Fig. 4*C*), no main effect of DREADD (*F*_(1,35)_ = 1.7; *p* = 0.2) and no stress × DREADD interaction (*F*_(1,35)_ = 3.9; *p* = 0.057). Planned comparisons revealed a significant reduction in immobility duration in the CVS hM4Di group compared with CVS Control (*p* = 0.02) and No CVS hM4Di group (*p* = 0.0009). There was a significant main effect of stress (*F*_(1,35)_ = 9.5; *p* = 0.004^g^; Fig. 4*C*) and time (*F*_(9,315)_ = 156.4; *p* < 0.0001^g^) on immobility duration but no interaction effects were observed among the three groups time × stress × DREADD (*F*_(9,315)_ = 1.1; *p* = 0.4). Planned comparisons revealed a significant decrease in immobility in the CVS hM4Di group at 2-, 3-, 4-, and 8-min time points compared with No CVS hM4Di group (*p* = 0.00009, *p* = 0.005, *p* = 0.04, and *p* = 0.007, respectively; Fig. 4*D*) and at the 3-min time point compared with the CVS Control group (*p* = 0.03; Fig. 4*D*).

Chronic PV IN inhibition had no effect on locomotor activity, demonstrated by no significant difference in total distance traveled (*t* = 0.74; df = 18; *p* = 0.46; Extended Data Fig. 4-1*A*) or velocity (*t* = 0.75; df = 18; *p* = 0.47; Extended Data Fig. 4-1*B*) demonstrating behavioral effects were not confounded by locomotor deficits. Control experiments performed on a separate group of animals to determine effects of chronic CNO in FST behavior showed no significant difference in immobility or swimming duration. Two-way ANOVA of total immobility duration did not show a significant main effect of stress (*F*_(1,28)_ = 0.81; *p* = 0.38; Extended Data Fig. 4-2*A*) no main effect of DREADD (*F*_(1,28)_ = 0.07; *p* = 0.8) and no stress × DREADD interaction (*F*_(1,28)_ = 0.13; *p* = 0.72). Two-way ANOVA of total swimming duration did not show a significant main effect of stress (*F*_(1,28)_ = 0.86; *p* = 0.36; Fig. 4-2*B*), no main effect of DREADD (*F*_(1,28)_ = 0.16; *p* = 0.69), and no stress × DREADD interaction (*F*_(1,28)_ = 0.11; *p* = 0.74).

### Chronic inhibition of PV INs during CVS: Impact on Fos induction by FST

**Figure 5.**
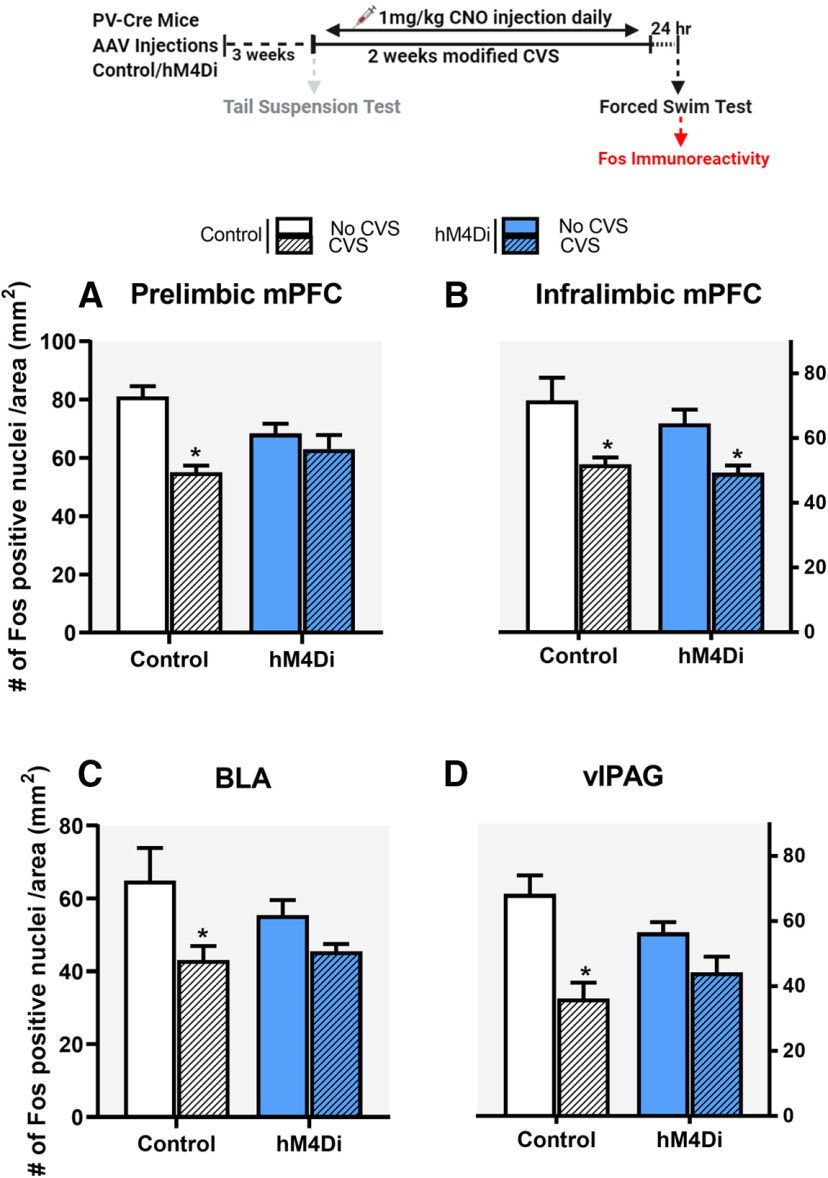
Fos immunoreactivity in the PrL, IL, BLA, and vlPAG. Chronic inhibition of PV INs in the IL mPFC during CVS, attenuated CVS-mediated reduction in Fos expression in the PrL, BLA, and vlPAG, respectively (***A***, ***C***, ***D***) but did not prevent CVS-mediated reduction in Fos expression in the IL cortex (***B***) following FST. Values are presented as mean ± SEM; *n* = 7–10 per group; * indicates significant result *p* < 0.05 *post hoc* (***A***) and planned comparisons (***C***, ***D***) compared with respective No CVS Control groups. PrL, IL, BLA, and vlPAG stands for prelimbic, infralimbic, basolateral amygdala and ventrolateral periaqueductal grey, respectively. Extended Data Figure 5-1 demonstrates Fos expression in various other brain regions where no significant effect of PV IN modulation was observed.

To test for Fos activation, animals were perfused after FST and brains were collected to analyze neuronal activation in brain regions typically activated by stress. We observed significant reduction in Fos induction in the CVS Control group compared with No CVS Control group, in the PrL, IL, BLA, and vlPAG. Inhibition of PV INs during CVS attenuated the reduction in Fos expression caused by CVS in the PrL, BLA, and vlPAG but not in the IL. Analysis of the PrL revealed a significant main effect of stress (*F*_(1,30)_ = 17.5; *p* = 0.0002 h; Fig. 5*A*) and a significant stress × DREADD interaction (*F*_(1,30)_ = 7.7; *p* = 0.009^h^). *Post hoc* analysis using Tukey’s test revealed a significant reduction in Fos expression in the CVS Control group (*p* = 0.0003), which was attenuated by chronic PV IN inhibition in the CVS hM4Di group (*p* = 0.75). There was a significant main effect of stress only (*F*_(1,27)_ = 21.2; *p* < 0.0001^i^; Fig. 5*B*) on Fos expression in the IL. There was a significant main effect of stress in the BLA (*F*_(1,20)_ = 12.4; *p* = 0.004^j^; Fig. 5*C*) and vlPAG (*F*_(1,17)_ = 20.5; *p* < 0.0001^k^; Fig. 5*D*) as well, with planned comparisons revealing significant reduction in Fos expression in the CVS Control group (*p* = 0.008 and *p* = 0.0009 in BLA and vlPAG, respectively) that was attenuated by chronic PV IN inhibition in the CVS hM4Di group (*p* = 0.75 and *p* = 0.08 in BLA and vlPAG, respectively). Analysis of Fos protein expression in the lateral septum (LS), anterior and ventral bed nucleus of the stria terminalis (BNST) and dorsolateral PAG (dlPAG) showed no significant treatment effects of PV IN inhibition, demonstrating those regions were not affected by PV IN modulation (Extended Data Fig. 5-1).

10.1523/ENEURO.0423-19.2020.f5-1 Extended Data Figure 5-1 Fos protein expression in several brain regions following CVS. Figure depicts Fos protein expression in lateral septum, anterior and posterior ventral BNST, and dlPAG. No significant treatment effects of PV IN inhibition was observed in any of the above-mentioned brain regions. Values represent mean ± SEM, *n* = 7–10 per group. Download Figure 5-1, TIF file.

### Chronic inhibition of PV INs during CVS: impact on somatic measurements

**Figure 6.**
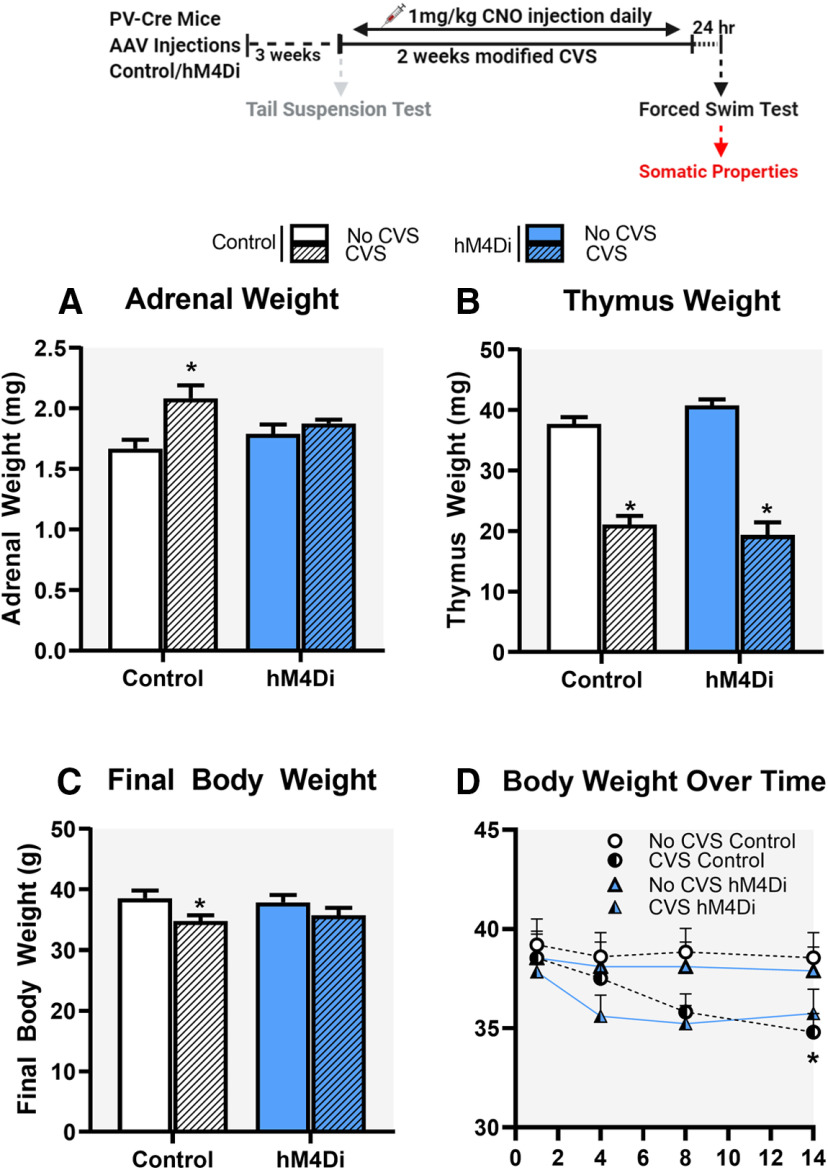
Impact of chronic stress on organ and body weights. Chronic inhibition of PV INs in the IL mPFC during CVS, attenuated CVS mediated increases in adrenal gland weight (***A***) and attenuated CVS mediated decreases in body weight (***C***, ***D***). No change in CVS-induced decreases in thymus weight was observed following PV IN inhibition (***B***). Data are presented as absolute organ and body weights. Values represent mean ± SEM; *n* = 9–10 per group; * indicates planned comparisons significant effect *p* < 0.05 versus corresponding No CVS Control groups.

Organs and body weights were used to assess somatic effects of CVS. Adrenal gland hypertrophy and/or thymic atrophy are often observed following chronic stress and are used as indicators of repeated/chronic hypothalamic pituitary adrenal (HPA) axis activation. In our experiments, there was a main effect of stress (*F*_(1,35)_ = 11.2; *p* = 0.002^l^; Fig. 6*A*) and a significant stress × DREADD interaction (*F*_(1,35)_ = 4.8; *p* = 0.035^l^) on adrenal weights. *Post hoc* analysis using Tukey’s test revealed that CVS Control group had significantly increased adrenal weight compared with No CVS Control group (*p* = 0.002), which was attenuated by CVS hM4Di when compared with No CVS hM4Di group (*p* = 0.84). There was a main effect of stress on thymus weight (*F*_(1,35)_ = 161.4; *p* < 0.0001^m^; Fig. 6*B*), with no effect of DREADD (*F*_(1,35)_ = 0.2; *p* = 0.7) or stress × DREADD interaction (*F*_(1,35)_ = 57; *p* = 0.1).

Decreased body weight gain is observed following chronic mild stress exposure in rodents (Ghosal et al., 2017). We observed a significant main effect of stress on final body weight (*F*_(1,35)_ = 6.6; *p* = 0.01^n^; Fig. 6*C*) with no effect of DREADD (*F*_(1,35)_ = 0.2; *p* = 0.6) or stress × DREADD interaction (*F*_(1,35)_ = 0.006; *p* = 0.9), consistent with known effects of CVS on body weight gain. Planned comparisons revealed final body weight in CVS group to be significantly lower than No CVS Control group (*p* = 0.03), which was attenuated by CVS hM4Di when compared with No CVS hM4Di group (*p* = 0.08; Fig. 6*C*). Two-way repeated measures ANOVA of body weight over time during the 14 d CVS paradigm showed a main effect of stress (*F*_(1,35)_ = 5.3, *p* = 0.03°) and time (*F*_(3,108)_ = 4.5; *p* = 0.005°; Fig. 6*D*). Planned comparisons revealed body weight in CVS Control group to be significantly lower than No CVS Control only on day 14 (*p* = 0.03; Fig. 6*D*). Control experiments to determine effects of chronic CNO on body weight showed no significant difference. Two-way ANOVA of total body weight showed a significant main effect of stress (*F*_(1,28)_ = 18.6; *p* = 0.002; Fig. 4-2*C*), no main effect of treatment (*F*_(1,28)_ = 0.41; *p* = 0.52) and no stress × treatment interaction (*F*_(1,28)_ = 0.21; *p* = 0.65). Planned comparisons revealed body weight in both Saline and CNO CVS groups to be significantly lower than respective No CVS controls (*p* = 0.04 and *p* = 0.01; Fig. 6*D*)

## Discussion

Our studies support a role for IL GABAergic PV INs in stress-mediated behavioral and somatic phenotypes. Using inhibitory DREADDs to inhibit the activity of PV INs during a modified CVS paradigm, we have established a causal role of IL PV INs in initiating and coordinating coping strategies and somatic outcomes in response to stress. Inhibition of PV IN during the CVS resulted in an increase in active coping strategy during FST, suggestive of dynamic behavioral remodeling during an aversive challenge. Chronic stress-induced behavioral alterations were accompanied by changes in neuronal activation patterns quantified by Fos expression following FST. Chronic PV IN inhibition attenuated CVS-induced reductions in Fos expression in PrL, BLA, and vlPAG, indicating that inhibition of PV INs mitigates the impact of chronic stress on stress regulatory brain regions. PV IN inhibition during CVS also attenuated CVS-induced adrenal hypertrophy and body weight loss, further suggesting that PV IN signaling during CVS might be playing a role in somatic effects of chronic stress. Interestingly, PV IN inhibition during the TST, which was the first stressor in the modified CVS paradigm, resulted in an increase in passive coping and a decrease in active coping in stress naive animals. This finding suggests unique mechanisms of PV IN plasticity following acute versus chronic stress paradigms and also implies that an optimum level of prefrontal PV IN activity is important to maintain prefrontal E/I balance so that PFC can respond appropriately to stress. Overall, the data indicate that PV INs play a role in inhibiting IL output during chronic stress, suggesting a potential role in driving ventromedial PFC hypofunction. Exploring the role of PV INs in stress-related disorders is an important research direction for future experiments.

GABAergic PV INs are well positioned to provide strong, fast-spiking inhibitory signals to pyramidal projection neurons in the PFC and reduce network excitability, and therefore could be contributing to chronic stress-mediated hypoactivity (Winkelmann et al., 2014; Tremblay et al., 2016; Safari et al., 2017). Our data suggest that PV INs play an important role in chronic stress-mediated inhibition of the IL. Chronic inhibition of IL PV INs during CVS resulted in increased active and decreased passive coping behaviors in FST. A switch to active coping can be interpreted as an adaptive strategy to deal with chronic stress, and drugs that are effective antidepressants in humans typically promote active coping styles and reduce passive coping in the FST in mice (Porsolt et al., 1977; Martí and Armario, 1993). GABA receptor antagonists have been shown to have antidepressant and anxiolytic properties (Bhutada et al., 2010; Mehta et al., 2017; Zanos et al., 2017; Samad et al., 2018). It is known that antidepressants such as fluoxetine and ketamine reduce PV expression in the PFC (Ohira et al., 2013; Zhou et al., 2015; Page and Coutellier, 2019). Moreover, preventing the reduction in PV IN activity leads to loss of antidepressant efficacy, further suggesting that reduced activity of PV INs might be playing a role in therapeutic efficacy of antidepressants (Zhou et al., 2015; Page and Coutellier, 2019). Therefore, based on prior studies and our findings, inhibition of PV INs during chronic stress may lead to more adaptive stress coping strategies and reverse some of the behavioral deficits associated with chronic stress. It is important to note that the effects observed in FST are because of PV IN inhibition and not because of any changes in locomotor activity (Extended Data Fig. 4–1). Additionally, we did not detect any effects on FST because of chronic dosing of CNO alone, suggesting that repeated CNO dosing did not alter stress coping behavior (Extended Data Fig. 4–2).

Our experiments revealed that inhibition of PV INs in the IL during stress can attenuate chronic stress-induced decreases in Fos expression in key stress regulatory regions such as the PrL, BLA, and vlPAG following FST (Keedwell et al., 2005; Berton et al., 2007; Vialou et al., 2014). Since we cannot verify direct PV IN modulation of IL projections to these regions, we cannot exclude the possibility that reversal of CVS-related inhibition of Fos induction is because of actions of the IL through other projection systems. Nonetheless, the data suggest that PV IN inhibition reduces inhibitory effects of CVS on IL outflow, permitting drive of downstream structures known to participate in physiological reactivity and stress coping behavior (Maier and Watkins, 2010). Notably, this includes the neighboring PrL, which is not targeted by our DREADD injections and thus has Fos excitability modulated by cortico-cortical connections. Involvement of the PrL is consistent with its prominent role in mediation of coping behavior (Fiore et al., 2015; Johnson et al., 2019; Molendijk and de Kloet, 2019).

PV IN modulation did not prevent CVS-induced changes in Fos in the IL following FST. The Fos data in IL represents an overall change in neuronal activity regardless of where the neurons project to. Thus, it is possible that PV IN modulation might be altering neural activity only in specific circuits, such as the IL-PrL or IL-BLA connections. Indeed, prior studies indicate that neurons projecting from IL to BLA receive strong innervation via PV INs (Marek et al., 2018). Additionally, activity of cortical PV INs is essential for microcircuit operations that correlate with behavioral events (Isomura et al., 2009; Kvitsiani et al., 2013; Kim et al., 2016). Thus PV INs might be affecting PFC microcircuits, thereby acting as a functional unit to synchronize the flow of information between IL-PrL (Courtin et al., 2014b; Kepecs and Fishell, 2014). Therefore, it will be important in the future to explore PV IN mediated plasticity in the specific circuits showing reduced Fos expression following PV IN modulation. It has been shown that chronic inhibition of neurons may also lead to plastic changes, resulting in rebound firing and enhanced excitability/hyperactivity when inhibition is removed (Stemmler and Koch, 1999; Sokolova and Mody, 2008; Wiegert et al., 2017; Purohit et al., 2018). Thus, it is also possible that removal of chronic inhibition of PV INs following CVS might have resulted in hyperactivity of PV INs, leading to reduced Fos expression in IL. CVS also causes a reduction in Fos on exposure to acute stress (Ostrander et al., 2009; Moench et al., 2019). Therefore, it is possible that the combined effect of rebound firing and CVS might be masking the Fos effects of chronic PV IN inhibition in the IL.

Repeated inactivation of PV INs during stress attenuated the CVS-induced increase of adrenal weight. The adrenals are highly sensitive to repeated stress, and it is believed that increased adrenal size is linked to cumulative increases in adrenocorticotropic hormone (ACTH) secretion (Ulrich-Lai et al., 2006). Blockade of adrenal hypertrophy suggests that PV INs participate in control of the central limb of HPA axis activation and provides additional confirmation of cumulative efficacy of chronic PV IN inhibition in control of stress endpoints. In contrast to the adrenals, CVS caused equivalent decreases in thymus weight, suggesting either sensitization of glucocorticoid sensitivity or enhanced autonomic activation by CVS, presumably mediated by mechanisms independent of PV INs. Chronic inhibition of PV INs also attenuates CVS-induced reduction in final body weight, suggesting PV IN signaling might also be playing a role in CVS mediated body weight effects.

As part of our design, we assessed the impact of IL PV IN inhibition acutely following the first stressor in our CVS regimen, the TST, which allows for behavioral readouts (duration of struggling, immobility and latency to immobility). Acute inhibition of IL PV INs resulted in decreased active coping (struggling) and increased passive coping (immobility) in the TST. Our data are consistent with a prior study indicating that reduced excitatory synaptic drive onto PV INs is linked to increased stress susceptibility and enhanced helplessness behavior (Perova et al., 2015). These data indicate that PV INs may play a role in driving active coping responses, when an animal with no history of prior stress is exposed to a novel acute stressor such as the TST. Together, these studies suggest that activation of PV INs is required for coping responses to acute stress.

Our results with acute PV IN inhibition are in contrast to the results seen in the FST after chronic PV IN inhibition during a two-week CVS exposure. These data indicate different roles for these neurons in acute versus chronic stress adaptations. Our finding of divergent effects of IN function in PFC is in line with previous studies showing opposing effects on emotionality in acute versus chronic somatostatin (SST) IN inhibition in the PFC (Soumier and Sibille, 2014) and on auditory information processing in acute versus chronic IN inhibition in the auditory cortex (Seybold et al., 2012). Our data suggest that distinct neuronal ensembles and brain circuitry may be involved in modulating acute versus chronic stress-mediated behavioral outcomes. It is also possible that chronic stress may result in plastic changes in the same neuronal ensemble recruited by acute stress, leading to differences in stress response. However, it is not known what specific plasticity in the neural network underlies the emergence of opposing phenotypes following chronic stress and therefore further studies are needed to investigate the mechanisms. Moreover, to fully understand the differences observed here with acute versus chronic stress PV IN modulation, it would be beneficial to determine whether chronic stress is able to modify the potentiating effect of acute inactivation on active coping responses in the TST.

Our acute and chronic PV IN inactivation data also suggest that an optimum level of E/I balance needs to be maintained in the PFC for it to function appropriately and PV INs play a crucial role in maintaining that balance (Ferguson and Gao, 2018). Disruption of the balance can lead to abnormal behavioral endpoints and psychiatric illnesses (Selten et al., 2018; Berg et al., 2019; Page and Coutellier, 2019). Our data on TST agree with prior reports suggesting that reducing activity of PV INs acutely under baseline conditions can lead to a more stress vulnerable passive coping response which might be because of an over excitation of the PFC (Perova et al., 2015). On the other hand, chronic stress can disrupt the E/I balance leading to over inhibition by increasing the activity of PV INs, resulting in maladaptive behavioral outcomes (Page et al., 2019; Page and Coutellier, 2019). The current approach is designed to inhibit the over activation of PV INs in the context of chronic stress exposure. Indeed, our results show that inhibiting the activity of PV INs during stress leads to a more active coping response and attenuates CVS mediated alterations in Fos activation in brain regions controlling emotionality as well as attenuating the effects on body and organ weights in response to CVS.

There are a few caveats to the present study that must be considered in the interpretation of the data. First, the modified CVS paradigm used in this study did not produce the typical behavioral effects seen in FST when using alternative CVS procedures (Ghosal et al., 2017; Wohleb et al., 2018). Initially, we thought that older age and chronic injection stress (the later described previously by Moghaddam and Bolinao, 1994), might have resulted in high rates of immobility in control animals leading to a ceiling effect, hence reducing the window to detect an increase in immobility typically observed after CVS exposure. However, additional control experiments showed CVS did not affect FST behavior in younger mice (Extended Data Fig. 4–3). Additional experiments in control mice showed chronic injection stress and single housing also did not affect behavior in FST (Extended Data Fig. 4-3). These results further lead us to conclude that the lack of effect on FST is because of the exclusion of overnight and extended periods of stress in the modified CVS paradigm in our study. Nevertheless, the paradigm resulted in somatic effects leading to reduced body weight and adrenal hypertrophy, indicating that sustained HPA axis drive only occurred in the CVS group. Moreover, our CVS procedure also reduced Fos activation in stress regulatory brain regions, demonstrating alterations in neuronal activation typically observed following CVS (Ostrander et al., 2009; Moench et al., 2019). Second, although our injection site was primarily in the IL, there was some spread into the PrL and DP area of the cortex (∼28% of cells). Third, this study was conducted only in male mice because prior research showed CVS mediated alteration in inhibitory synaptic drive in the IL of males (McKlveen et al., 2016, 2019). Because PV IN modulation may have sex specific effects (Shepard et al., 2016; Page et al., 2019), it would be important to examine the effects of PV IN modulation during stress in females. Fourth, in this study PV INs were inhibited during the 14 d of CVS. The degree to which the efficacy of DREADD-mediated inhibition might vary over the course of the 14-d treatment paradigm remains unclear and will require additional study. Finally, in this study we euthanized animals after exposure to one behavioral paradigm (FST) to obtain the anatomic Fos expression dataset to a novel stressor following CVS. Chronic stress can be characterized by cellular and behavioral changes spanning multiple interconnected neural network adaptations which were not explored in our current study. In order to get a clear representation of how PV IN modulation during stress is affecting emotionality, additional behaviors may be worth exploring in follow-up experiments.

Taken together, our data are consistent with a causal role of IL PV INs in initiating and coordinating coping strategies and physiological outcomes in response to stress. Chronic stress-mediated hypoactivity and aberrant behavioral responses may be mediated partly via plastic changes in PV IN function and may play a role in stress-related pathologies (e.g., depression and PTSD). Our data indicate that chemogenetic inhibition of PV INs during chronic stress, which reduces PV-initiated inhibition in the context of each individual stressor experience, may block or attenuate inhibition of glutamatergic neurons. In this case, maintenance of inhibitory synaptic inputs onto glutamatergic IL projection neurons is sufficient to attenuate some but not all behavioral and physiological consequences of chronic stress exposure, including decreased passive (immobility) and increased active coping behaviors (swimming) in the FST, attenuating CVS effects on reduction in neuronal Fos activity, adrenal hypertrophy, and body weight loss. Our findings suggest that reducing the activity of PV INs in the PFC during chronic stress may facilitate output of prefrontal neurons and could provide therapeutic benefits for stress-related disorders.

In conclusion, this study provides support that PV INs play a role in chronic stress-mediated coping behaviors and physiological phenotypes. Furthermore, the study adds to the current knowledge regarding possible mechanisms of hypoactivity of the PFC and how PV INs may be involved in driving chronic stress-related pathologies. The study also highlights opposing effects of acute and chronic PV IN inhibition, indicating different underlying mechanisms involved in acute versus chronic stress paradigms. Overall, this study shows that reducing PV IN activity to promote prefrontal output may be an effective treatment strategy for stress-related illnesses.

## Synthesis

Reviewing Editor: Carmen Sandi, Swiss Federal Institute of Technology Decisions are customarily a result of the Reviewing Editor and the peer reviewers coming together and discussing their recommendations until a consensus is reached. When revisions are invited, a fact-based synthesis statement explaining their decision and outlining what is needed to prepare a revision will be listed below. The following reviewer(s) agreed to reveal their identity: Francesco Papaleo.

This ms. constitutes an interesting study on the role of prefrontal cortical interneurons in stress-related circuitry and coping behaviors. In this revised version, the authors have provided thoughtful and detailed answers to the previous review and substantial and very valuable modifications that have greatly improved the manuscript. The two reviewers and the review editor consider that this revised version constitutes an important addition to the literature, and only have three minor requests for further consideration:

-Avoidance of the interpretation that chronic prefrontal parvalbumin interneuron (PV IN) inactivation, per se, can reverse passive coping responses (i.e., comparison of TST vs. FST in non-CVS DREADD). While the authors are correct in pointing out that the CNO is not on board after chronic administration over the 14d prior, there is little alternative to account for the fact that chronic CNO administration given for 14 continuous days prior to a stress coping test has different effects than a single acute CNO injection just prior. It is appreciated that the authors wish to focus on the interactive effects of PV IN inactivation under chronic stress, yet the reader will be left wondering about the contribution of inactivating PV IN neurons under baseline conditions. A further discussion on these issues would improve the manuscript.

-The argument against any need to physiologically validate the DREADDs is specious. The authors contend that if they were to not observe any effect of CNO on the 14th day of administration, then it doesn't necessarily mean that the CNO didn't work through the earlier course of its daily administration, and that the only correct way would be to test CNO's effects over all 14 days to see where the response drops off. Yet, this idea doesn't account for the fact that a lack of an effect, or even compensatory increase, observed on day 14 would impel a different interpretation than the current one offered.

- Concerning the first major concern from the previous review regarding chronic stress effects on the FST, it would be relevant to include the additional data reported to the reviewers, as well as the accompanying explanation, in the ms.

## Author Response

Responses to reviewers after 2nd revision

Thank you very much for the time and effort devoted to the revision of our manuscript titled "Chemogenetic Inhibition of Infralimbic Prefrontal Cortex GABA-ergic Parvalbumin Interneurons Attenuates the Impact of Chronic Stress in Male Mice"

(eN-NWR-0423-19R1). We appreciate the comments and suggestions made by the editor and the expert reviewers. We are glad to know the editor and the reviewers found the manuscript to be greatly improved from the previous version. We have attached our point by point response to the minor revisions requested.

Minor revisions: (Changes in manuscript text highlighted in green)

1. Avoidance of the interpretation that chronic prefrontal parvalbumin interneuron (PV IN) inactivation, per se, can reverse passive coping responses (i.e., comparison of TST vs. FST in non-CVS DREADD). While the authors are correct in pointing out that the CNO is not on board after chronic administration over the 14d prior, there is little alternative to account for the fact that chronic CNO administration given for 14 continuous days prior to a stress coping test has different effects than a single acute CNO injection just prior. It is appreciated that the authors wish to focus on the interactive effects of PV IN inactivation under chronic stress, yet the reader will be left wondering about the contribution of inactivating PV IN neurons under baseline conditions. A further discussion on these issues would improve the manuscript.

In discussion of the acute effects of PV IN inactivation it has been stated 'Moreover, in order to fully understand the differences observed here with acute vs chronic stress PV IN modulation, it would be beneficial to determine whether chronic stress is able to modify the potentiating effect of acute inactivation on active coping responses in the TST.' Furthermore, discussion on the contribution of inactivating PV IN under baseline conditions has been updated. The discussion is based on the importance of maintaining the prefrontal excitatory- inhibitory balance which is crucial for appropriate prefrontal drive.

2. The argument against any need to physiologically validate the DREADDs is specious. The authors contend that if they were to not observe any effect of CNO on the 14th day of administration, then it doesn't necessarily mean that the CNO didn't work through the earlier course of its daily administration, and that the only correct way would be to test CNO's effects over all 14 days to see where the response drops off. Yet, this idea doesn't account for the fact that a lack of an effect, or even compensatory increase, observed on day 14 would impel a different interpretation than the current one offered.

Discussion has been updated to state that the degree to which the efficacy of DREADD-mediated inhibition might vary over the course of the 14 day treatment paradigm remains unclear and would require additional study.

3. Concerning the first major concern from the previous review regarding chronic stress effects on the FST, it would be relevant to include the additional data reported to the reviewers, as well as the accompanying explanation, in the ms.

Additional data and accompanying explanation on chronic stress effects on the FST has been added to the manuscript. The rationale for using the modified CVS is included in the 'Methods' and the additional data and accompanying discussion of age, injection stress and housing is included in the 'Discussion' and added to extended figure 4-3.

Responses to reviewers after 1st revision

Thank you very much for the time and effort devoted to the revision of our manuscript titled "Chemogenetic Inhibition of Infralimbic Prefrontal Cortex GABA-ergic Parvalbumin Interneurons Attenuates Chronic Stress Adaptions in Male Mice"(eN-NWR-0423-19). We appreciate the comments and suggestions made by the editor and the expert reviewers. We have carefully analyzed the comments and concerns, performed additional control experiments requested and have addressed the questions and provided clarification to the reviewer comments.

Major Concerns:

1. An important issue is the lack of effects of the chronic variable stress (CVS) protocol used on a key output test used for the study, the forced swim test (FST). Whereas the study aimed at testing whether increased activity in mPFC PV neurons underlies CVS-mediated prefrontal hypofunction, and its rescue by inactivating mPFC-PV neurons using DREADDs; the fact that the CVS had no effect in FST behavior precludes making such an interpretation. Instead, the data show that PV inactivation decreases immobility/ increases swimming as a function of CVS exposure. Although the Discussion section addresses this caveat (addressed as well in the limitation of the study; but then a group of younger mice should have been checked as well), the more fundamental issue would be addressed by repeating the study to see if the authors are able produce the needed stress-phenotype, using a more robust repeated stress model, or selecting a different endpoint that is sensitive to their CVS model.

One of the possible reasons for the lack of effect of CVS on FST was due to the fact that the CVS protocol used in this study did not include extended stress periods and overnight stressors that are typically used in CVS paradigms (Ghosal et al., 2017; Ostrander et al., 2009; Wohleb et al., 2018). This was done to ensure that CNO was on-board throughout the stressor (Baldessarini et al., 1993; Guettier et al., 2009; Jendryka et al., 2019; Duncan A.A. MacLaren et al., 2016). CNO has been shown to reach maximal plasma levels 30 minutes post i.p injection and has been shown to rapidly clear from plasma in rodents (Baldessarini et al., 1993; Guettier et al., 2009; Jendryka et al., 2019; Duncan A.A. MacLaren et al., 2016). We chose intraperitoneal (i.p.) injections for CNO delivery to effectively control the dose and timing of DREADDs during our stress paradigm. Additional control experiments (please see below) revealed that age of the animal was not playing a role in the FST phenotype, further suggesting that the modified CVS protocol likely lead to the effects observed. We chose FST as an endpoint since previous studies have shown that PFC PV IN modulation in males caused deficits in stress coping behavior in a learned helplessness model (Perova et al., 2015). Change in PV IN expression was also thought to underlie depression-like behavior in FST in male mice (Shepard et al., 2016a). Moreover we wanted to explore stress coping behaviors since we know anxiety like behavioral domains are not affected by PV IN modulation in males (Page et al., 2019; Shepard et al., 2016b).

Although there was a lack of CVS effect on behavior, we clearly saw that CVS resulted in somatic effects leading to reduced body weight and adrenal hypertrophy indicating that sustained HPA axis drive only occurred in the CVS group. The modified CVS also reduced Fos activation in brain regions, demonstrating alterations in neuronal activation typically observed in CVS animals following an acute stress (Moench et al., 2019; Ostrander et al., 2009). PV IN inhibition during CVS altered Fos activation patterns and somatic effects associated with CVS. PV IN inhibition during CVS also altered coping behavior in FST. Moreover the effects observed in FST were opposite to that in the tail suspension test implying different plasticity mechanisms following acute vs chronic stress paradigms. For the above reasons we believe our findings are important and will add to the growing literature evidence of the role of PV INs in stress related illnesses.

Additional Control Experiments (Data not included in the paper)

Initially we thought the high rates of immobility in FST in our control animals were due to old age and chronic injection stress (the later described previously by (Moghaddam and Bolinao, 1994)), which might have resulted in a ceiling effect, reducing the window to detect an increase in immobility typically observed after CVS exposure. As suggested by the reviewers, we have checked younger mice and found no significant effect of our CVS on FST (Figure below). Additionally, we performed experiments in control mice to determine the effects of chronic injection stress and single housing, none of which affected behavior in FST. This led us to further conclude that the lack of effect on FST is due to the use of the modified CVS paradigm in our study.

2. There are several methodological issues that need to be addressed. 1) It is important to show that the virus was selectively injected and only infected PV neurons in the infralimbic without affecting the adjacent prelimbic cortex. To this end, previous evidence from the literature indicates that the injection should be done diagonally to avoid spreading of the virus throughout the injector (e.g. Mukherjee and Caroni Nature Comm 2018). 2) It is important to report whether chronic CNO had any effects per se. Currently, the manuscript includes data from 4 mice treated with saline; this group would not be statistically sufficient to properly compare with the effects of chronic injections with CNO. We are also concerned that the manuscript seems to indicate that this group of saline-treated mice did not go through the different stress manipulations and, hence, would not be a proper control group, as CNO might interact with the stress manipulations affecting its behavioral effects. 3) It would be important to confirm (classically this is done with electrophysiological analyses) that CNO inhibited PV+ cells even after chronic treatment, as compensatory or desensitization mechanisms might occur following this manipulation.

1) Prior to initiation of this study, we had conducted a pilot study with 8 animals to optimize the viral load, injection volume and stereotactic coordinates that resulted in DREADD transfection specifically in the IL. We specifically chose serotype 2 of AAV since it has a small volume of transfection and mostly transduces cells near the injection site enabling us to target IL specifically (Burger et al., 2004; Cearley and Wolfe, 2006). Additionally, we made sure to take care of a few key points, now outlined in Materials and Methods section to ensure viral spread was mostly restricted to IL.

As suggested by the reviewers, we have now modified Figure 2 to show that virus was selectively injected and primarily transduced cells in the IL. Figure 2 depicts injection sites and viral spread from animals injected with the hM4Di DREADD. Viral spread was mapped onto its respective focal plane and bregma level by outlining the spread of the infection from confocal images onto corresponding brain atlas illustrations. The percentage of infected cells in IL was calculated by dividing the number of mCherry positive cells in IL by the total number of mCherry positive cells in a specific focal plane. Brain bregma coordinates used for IL cytoarchitecture were defined in the Franklin and Paxinos mouse brain atlas (3rd Edition) (Franklin and Paxinos, 2008). Figure 2 shows that about 72% of PV cells expressing DREADDs were in the IL with some minor spread to prelimbic area (above IL) and dorsal peduncular cortex (located ventral to IL).

2) As suggested by the reviewers, we performed control experiments using a 2x2 study design with stress and CNO as factors with n=8 animals per group. Our results show chronic CNO did not have behavioral effects on its own or when paired with CVS. Data is shown in extended figure 4-2.

3) Our planned electrophysiological experiment to determine if chronic CNO treatment caused desensitization of DREADD, was delayed due to Covid-19. Once activities resumed after 2.5 months, we tried to perform patch clamp electrophysiology. However, we were not able to obtain reliable recordings since obtaining healthy transfected cells from adult mouse cortex proved to be a challenge. We also thought that the results would not add much because, assuming that we do not see CNO effect on 14th day, that does not necessarily mean that CNO did not work and it does not tell us when CNO stopped working. For example, if CNO was efficacious on days 10th, 12th or 13th, then that would still be a significant duration of time during which PV INs were inhibited during CVS. The only way to tackle this is to perform electrophysiology everyday throughout the 14 days to see if and when the effect is lost.

Nevertheless, there is significant evidence in the literature that chronic DREADD manipulation does not lead to desensitization of receptors during the 14 day time frame used in this study (please see evidence a-c below). We also saw behavioral and physiological effects after 14 days of PV IN modulation, suggesting chronic CNO is efficacious in our study. Hence, we decided to go forward with the resubmission without the electrophysiology experiment.

a. Significant desensitization of DREADDs following chronic manipulations does not occur in vivo (Roman et al., 2016; Roth, 2016). Viral and transgenic expression methods express DREADDs at high levels, likely circumventing the problem of desensitization after repeated activation (Burnett and Krashes, 2016; Roth, 2016). Studies using prolonged CNO delivery have not reported difficulty with repeated activation of DREADDs (Krashes et al., 2013; Zhan et al., 2013).

b. Chronic (month-long) CNO treatment inhibited hM4Di-infected neurons without altering baseline neuronal excitability in PV-Cre mice. (Bartos et al., 2017). Behavioral effects with inhibitory hM4Di-DREADD is seen after chronic CNO treatment for 15 (Phillips et al., 2019) and 21 days (Soumier and Sibille, 2014) suggesting no desensitization of hM4Di receptors.

c. Twice daily 1mg/kg CNO treatment in PV-Cre mice for 14 days was able to activate hM3Dq DREADD (Stedehouder et al., 2018). 0.5mg/kg CNO application for 21 days was able to activate hM3Dq DREADD in mPFC of PV-Cre mice (Page et al., 2019). Other studies also confirm that 1mg/kg CNO i.p for 14 days also able to activate hM3Dq (Cheng et al., 2019).

3. Need for additional control groups: The study reports that chronic inactivation of mPFC-PV neurons decreases immobility in TST/FST-like tests; whereas acute PV inactivation increases immobility on TST (e.g., Fig. 3B), chronic PV inactivation reverses this effect to levels no different from CNO controls (e.g., FST data, Fig 4B, compare white bars in control vs. hM4Di). On the one hand, chronic PV inactivation may be restoring a control phenotype. Whereas its administration on the first day produces a stress-vulnerable phenotype, over time it balances out to a more normative response. While it is unclear if these studies would be resolved by, or need, additional experimentation, obvious controls that could help to clear up these uncertainties would involve administration a saline-only injected group, waiting to give CNO until after the CVS regimen has been completed, or testing whether the effects of activating PV neurons would bi-directionally modulate the effects observed here. Importantly, the effects found after acute and chronic PV inhibition might just be due to changes in locomotor function. These control tests are missing as well.

1) We have included locomotor data (total distance travelled and velocity) showing chronic inhibition of PV INs have no effect on locomotor activity (Figure 4-1). The locomotor activity was measured during exposure to a Y maze which was used as a stressor on day 12th of CVS regimen. CNO was applied 30 minutes before onset of stress. Additionally, previous studies in the past have shown that acute inhibition of PV INs in PFC had no effect on locomotor activity (Perova et al., 2015). Chronic excitation of PV IN in the PFC also does not affect locomotor activity (Page et al., 2019). Additionally a low dose of 1 mg/kg CNO in general has no effect on locomotor activity (D. A. A. MacLaren et al., 2016). Therefore, we have strong evidence to support that the effects in TST and FST are not due to changes in locomotor function.

2) We are not directly comparing or analyzing the TST and FST results and NOT making the conclusion that chronic PV IN is reversing the TST effects. We have stated the observed differences in discussion and said that this might imply that acute vs chronic stress may have different plastic mechanisms on PV IN and further supported that with evidence in the literature. The main purpose of our current experiment was to test whether inhibition of PV INs during the chronic stress regimen, could block the aggregate effect of repeated stress on subsequent coping behavior (FST used as a novel stressor). Therefore, no CNO was on board when FST was conducted. On the other hand, the effects of TST were due to acute inhibition of PV INs, where TST was the first stressor in the CVS paradigm. The TST data indicate that PV INs may play a role in driving active coping responses, when an animal with no history of prior stress is exposed to a novel acute stressor such as the TST.

The interpretation that "whereas its administration on the first day produces a stress-vulnerable phenotype, over time it balances out to a more normative response" is clarified and explained in greater detail in the discussion: An optimum level of E/I balance needs to be maintained in the PFC for it to function appropriately and PV INs play crucial role to maintain that balance (Ferguson and Gao, 2018). Disruption to the balance can lead to abnormal behavioral endpoints and psychiatric illnesses (Berg et al., 2019; Page and Coutellier, 2019; van Bokhoven et al., 2018). Our data on tail suspension test and prior studies have shown that reducing activity of PV IN can lead to a more "stress vulnerable" passive coping response which might be due to an "over excitation" of the prefrontal cortex (Perova et al., 2015). On the other hand, chronic stress has been shown to disrupt the E/I balance leading to "over inhibition" by increasing the activity of PV IN which leads to maladaptive behavioral outcome (Page et al., 2019; Page and Coutellier, 2019). The current approach is designed to inhibit the over activation of PV INs in the context of chronic stress exposure. Our results show that inhibiting the activity during CVS lead to a more active coping response, blunts CVS mediated alterations in Fos activation in brain regions controlling emotionality and alters somatic responses associated with CVS. Importantly, these effects were only observed in the hM4Di CVS group and not by just modulating PV IN in the hM4Di No CVS group.

3) We agree with the reviewer that it would be interesting to know what would happen if PV INs are inhibited after CVS mediated plasticity has already occurred in the interneurons, by administering CNO after CVS. Discussion has been updated to include this experiment as future direction.

4. The analysis of the Fos expression data raise a number of items needing clarification. The first issue concerns the fact that chronic PV inactivation had no effect on Fos expression in infralimbic cortex (IL). Additional concerns are raised regarding the interpretation that mPFC-PV inactivation attenuated CVS-induced decreases in Fos in several other regions (e.g., prelimbic, amygdala, periaqueductal gray). Here this lack of difference appears to be more due to the fact that chronic CNO treatment in controls was producing overall decreases in these circuits, not that inactivation of PV neurons was rescuing a CVS-effect or deficit as proposed in the manuscript.

1) PV IN modulation did not prevent CVS induced changes in Fos in the IL following FST. The Fos data in IL represents an overall change in neuronal activity irrespective of where the neurons are projecting to. Thus, it is possible that PV IN modulation might be altering neural activity only in specific circuits, such as the IL-Prelimbic (PrL) or IL-BLA connections. Indeed, studies have shown that neurons projecting from IL to BLA receive strong innervation by PV INs (Marek et al., 2018). Additionally, activity of cortical PV INs has been shown to be essential for microcircuit operations which correlates to behavioral events (Hoseok Kim et al., 2016; Isomura et al., 2009; Kvitsiani et al., 2013). Thus PV IN might be affecting PFC microcircuits, acting as a functional unit to synchronize the flow of information between IL-PrL, and hence PrL cortex was affected more due to PV IN modulation (Courtin et al., 2014; Kepecs and Fishell, 2014).

It has been shown that chronic inhibition of neurons may also lead to plastic changes, resulting in rebound firing and enhanced excitability/hyperactivity when inhibition is removed (Purohit et al., 2018; Sokolova and Mody, 2008; Stemmler and Koch, 1999; Wiegert et al., 2017). Thus, it is also possible that removal of chronic inhibition of PV INs following CVS might have resulted in hyperactivity of PV INs, leading to reduced Fos expression in IL. CVS also causes a reduction in Fos upon exposure to acute stress (Moench et al., 2019; Ostrander et al., 2009). Therefore, the combined effect of rebound firing and CVS might be masking the Fos effects of chronic PV IN inhibition in the IL.

2) In response to the second comment that chronic CNO treatment in controls was producing overall decreases in these circuits- it should be pointed out that all groups received CNO in this study design. Therefore, CNO effects on Fos are counterbalanced across groups. If the reviewer was referring to reduce Fos expression in the No CVS hM4Di group, it should be noted that the No CVS hM4Di group was not statistically significant from the No CVS Control virus group in any of the regions examined, therefore we cannot truly say that PV IN inhibition was causing decreases in these circuits.

Minor Concerns:

Abstract:

1) In the abstract, the following too general statement should be revised: "...that enhanced activity of parvalbumin (PV) expressing GABAergic interneurons (INs) play a role in chronic stress related pathologies." There are actually several "stress-related pathologies" where PV are supposed to be reduced and not enhanced. Statement revised to remove the term "stress-related pathologies" and updated to state 'prior evidence suggests that chronic stress leads to an increase in expression and activity of parvalbumin (PV) expressing GABAergic interneurons (INs) in the PFC.' We acknowledge that some studies report reduced PV activity following chronic stress, however those changes in PV were mainly observed in the hippocampus (Czeh et al., 2005; Rossetti et al., 2018). Several studies show increased GABA-ergic tone, enhanced PV expression and increased activity of PV INs in the PFC following chronic stress (McKlveen et al., 2016; Page et al., 2019; Page and Coutellier, 2019). Nonetheless, the differences in PV expression observed following stress could be due to the heterogeneity in stress protocols and duration of the chronic stress regimen used in the experiments. It is possible that during a 2 week chronic stress period there is an increase in PV activity (McKlveen et al., 2016; Page and Coutellier, 2019), and if stress is continued for longer periods of time such as 7 weeks, there is an eventual decrease in PV activity due to damage and PV cell loss (Rossetti et al., 2018).

2) The abstract sets the ground for enhanced PV activity; however, the study explores the effects of PV inhibition. To recapitulate prefrontal hypoactivity, activation of PV cells would be more appropriate. We know chronic stress leads to enhanced PV activity in the PFC. The purpose of the study was to determine if reducing PV activity during chronic stress, would be sufficient to prevent chronic stress related phenotypes. In other words, we are examining if enhanced PV activity during stress is "necessary" to engender the chronic stress phenotypes. We are not trying to recapitulate prefrontal hypoactivity or determine if PV activity is "sufficient" which would call for activating PV cells in the absence of stress. That is a different question and indeed an important one, and would be worth exploring in future studies.

3) The abstract refers to PFC in general or even prelimbic cortex, however the study focuses on the "infralimbic cortex". The term Infralimbic cortex has been added to the abstract : "In this study, the role of Infralimbic PFC PV INs in stress related phenotypes were explored.."

Introduction:

It is not clear and it important to state whether PFC hypofunctioning is a cause or consequence on the cited psychiatric disorders. And/or if PFC hypofunction is caused by stress or, viceversa, modulate the responses to stressful events. PFC hypofunction is a consequence of chronic stress and is thought to underlie stress related abnormalities and psychiatric disorders. This point is now highlighted in the introduction.

Methods and Results:

1) This statement in the results section seems misleading: "Inhibition of PV INs during CVS prevented the reduction in Fos expression caused by CVS in the PrL, BLA and vlPAG, but not in the IL." Indeed, the data show that PV inhibition reduce cFos expression in the No CVS group...preventing to see any further reduction in the CVS group. There was no significant effect of hM4Di vs control virus in the No CVS group in any of the regions examined. Therefore we cannot truly say that PV IN inhibition reduce cFos expression in the No CVS group. However, based on the reviewer comment, instead of saying prevented we now say that PV IN modulation attenuated the CVS effects.

2) How mice were housed during the stress manipulations? Were mice from different experimental groups housed together? All mice in both stress and control groups were single housed. Methods section updated to include the detail. We have done control experiments to show that single housing does not affect their behavior in FST( data not shown)

3) It is assumed that the transgenic mouse used have been characterized and published elsewhere. It would be important to include these references. References have been added in Materials and Section under the subheading Mice

4) In figure 2, explicit boundaries are drawn indicating that injection placements were in infralimbic mPFC. The authors should clearly state the cytoarcitectonic basis for this distinction. Figure 2 has been updated to show cytoarchitectonic basis of IL distinction.

5) In figure 2A, isn't mCherry supposed to be shown inverted and then flipped by Cre recombinase (similar to how hM4Di mCherry is shown right above)? That is correct. Figure was updated to show mCherry was flipped by Cre- please see Figure 1B

6) Please, indicate clearly whether the same animals subjected to TST were used later for FST? It may be interesting if any insight can be gleaned about treatment effects within-subjects. Same animals subjected to TST were used later for FST. Text updated to state that clearly under Forced Swim Test in Materials and Methods section. Additionally, we included study timeline above each figure which now shows that animals with TST also had FST. Given the difference in testing protocols, we felt that it was not appropriate to compare TST and FST data.

7) It may be easier for the reader if white and black color schemes are used in a consistent manner throughout the figures. For example, in Fig. 3, controls are in white and hM4Di in black, whereas in subsequent figures white represents the control (i.e., "no CVS") and black represents CVS, and control and hM4Di are both black and white. Color schemes have been changed. Animals in the CVS group had both TST and FST and color schemes have been updated to reflect that. Additionally, we included study timeline above each figure which now shows that animals with TST also had FST.

References:

Baldessarini RJ, Centorrino F, Huston Lyons D, Cohen BM, Flood JG, Volpicelli SA (1993) Tissue concentrations of clozapine and its metabolites in the rat. Neuropsychopharmacology 9:117-124.

Bartos M, Xia F, Richards BA, Tran MM, Josselyn SA, Takehara-Nishiuchi K, Frankland PW (2017) Parvalbumin-positive interneurons mediate neocortical-hippocampal interactions that are necessary for memory consolidation. Elife 6:1-25.

Berg L, Eckardt J, Masseck OA (2019) Enhanced activity of pyramidal neurons in the infralimbic cortex drives anxiety behavior. PLoS One 14:e0210949.

Burger C, Gorbatyuk OS, Velardo MJ, Peden CS, Williams P, Zolotukhin S, Reier PJ, Mandel RJ, Muzyczka N (2004) Recombinant AAV viral vectors pseudotyped with viral capsids from serotypes 1, 2, and 5 display differential efficiency and cell tropism after delivery to different regions of the central nervous system. Mol Ther 10:302-317.

Burnett CJ, Krashes MJ (2016) Resolving Behavioral Output via Chemogenetic Designer Receptors Exclusively Activated by Designer Drugs.

Cearley CN, Wolfe JH (2006) Transduction characteristics of adeno-associated virus vectors expressing cap serotypes 7, 8, 9, and Rh10 in the mouse brain. Mol Ther 13:528-537.

Cheng J, Umschweif G, Leung J, Sagi Y, Greengard P (2019) HCN2 Channels in Cholinergic Interneurons of Nucleus Accumbens Shell Regulate Depressive Behaviors Article HCN2 Channels in Cholinergic Interneurons of Nucleus Accumbens Shell Regulate Depressive Behaviors. Neuron 101:662-672.e5.

Courtin J, Chaudun F, Rozeske RR, Karalis N, Gonzalez-Campo C, Wurtz H, Abdi A, Baufreton J, Bienvenu TCM, Herry C (2014) Prefrontal parvalbumin interneurons shape neuronal activity to drive fear expression. Nature 505:92-96.

Czeh B, Simon M, Van Der Hart MGC, Schmelting B, Hesselink MB, Fuchs E (2005) Chronic stress decreases the number of parvalbumin-immunoreactive interneurons in the hippocampus: Prevention by treatment with a substance P receptor (NK1) antagonist. Neuropsychopharmacology 30:67-79.

Ferguson BR, Gao W-J (2018) PV Interneurons: Critical Regulators of E/I Balance for Prefrontal Cortex-Dependent Behavior and Psychiatric Disorders. Front Neural Circuits 12:37.

Ghosal S, Packard AEB, Mahbod P, McKlveen JM, Seeley RJ, Myers B, Ulrich-Lai Y, Smith EP, D'Alessio DA, Herman JP (2017) Disruption of Glucagon-Like Peptide 1 Signaling in Sim1 Neurons Reduces Physiological and Behavioral Reactivity to Acute and Chronic Stress. J Neurosci 37:184-193.

Guettier JM, Gautam D, Scarselli M, De Azua IR, Li JH, Rosemond E, Ma X, Gonzalez FJ, Armbruster BN, Lu H, Roth BL, Wess J (2009) A chemical-genetic approach to study G protein regulation of β cell function in vivo. Proc Natl Acad Sci U S A 106:19197-19202.

Hoseok Kim A, Wang X, Deisseroth K, Carlé Correspondence M, Kim H, Carlé M (2016) Prefrontal Parvalbumin Neurons in Control of Attention In Brief Prefrontal Parvalbumin Neurons in Control of Attention. Cell 164:208-218.

Isomura Y, Harukuni R, Takekawa T, Aizawa H, Fukai T (2009) Microcircuitry coordination of cortical motor information in self-initiation of voluntary movements. Nat Neurosci 12:1586-1593.

Jendryka M, Palchaudhuri M, Ursu D, van der Veen B, Liss B, Kätzel D, Nissen W, Pekcec A (2019) Pharmacokinetic and pharmacodynamic actions of clozapine-N-oxide, clozapine, and compound 21 in DREADD-based chemogenetics in mice. Sci Rep 9.

Kepecs A, Fishell G (2014) Interneuron cell types are fit to function. Nature.

Krashes MJ, Shah BP, Koda S, Lowell BB (2013) Rapid versus delayed stimulation of feeding by the endogenously released agRP neuron mediators GABA, NPY, and AgRP. Cell Metab 18:588-595.

Kvitsiani D, Ranade S, Hangya B, Taniguchi H, Huang JZ, Kepecs A (2013) Distinct behavioural and network correlates of two interneuron types in prefrontal cortex. Nature 498:363-366.

MacLaren D. A. A., Browne RW, Shaw JK, Krishnan Radhakrishnan S, Khare P, Espana RA, Clark SD (2016) Clozapine N-Oxide Administration Produces Behavioral Effects in Long-Evans Rats: Implications for Designing DREADD Experiments. eNeuro 3.

MacLaren Duncan A.A., Browne RW, Shaw JK, Radhakrishnan SK, Khare P, España RA, Clark SD (2016) Clozapine N-oxide administration produces behavioral effects in long-evans rats: Implications for designing DREADD experiments. eNeuro 3:219-235.

Marek R, Jin J, Goode TD, Giustino TF, Wang Q, Acca GM, Holehonnur R, Ploski JE, Fitzgerald PJ, Lynagh T, Lynch JW, Maren S, Sah P (2018) Hippocampus-driven feed-forward inhibition of the prefrontal cortex mediates relapse of extinguished fear. Nat Neurosci 21:384-392.

McKlveen JM, Morano RL, Fitzgerald M, Zoubovsky S, Cassella SN, Scheimann JR, Ghosal S, Mahbod P, Packard BA, Myers B, Baccei ML, Herman JP (2016) Chronic Stress Increases Prefrontal Inhibition: A Mechanism for Stress-Induced Prefrontal Dysfunction. Biol Psychiatry 80:754-764.

Moench KM, Breach MR, Wellman CL (2019) Chronic stress produces enduring sex- and region-specific alterations in novel stress-induced c-Fos expression. Neurobiol Stress 10.

Moghaddam B, Bolinao ML (1994) Glutamatergic antagonists attenuate ability of dopamine uptake blockers to increase extracellular levels of dopamine: Implications for tonic influence of glutamate on dopamine release. Synapse.

Ostrander MM, Ulrich-Lai YM, Choi DC, Flak JN, Richtand NM, Herman JP (2009) Chronic stress produces enduring decreases in novel stress-evoked c- fos mRNA expression in discrete brain regions of the rat. Stress 12:469-477.

Page CE, Coutellier L (2019) Prefrontal excitatory/inhibitory balance in stress and emotional disorders: Evidence for over-inhibition. Neurosci Biobehav Rev 105:39-51.

Page CE, Shepard R, Heslin K, Coutellier L (2019) Prefrontal parvalbumin cells are sensitive to stress and mediate anxiety-related behaviors in female mice. Sci Rep 9.

Perova Z, Delevich K, Li B (2015) Depression of excitatory synapses onto parvalbumin interneurons in the medial prefrontal cortex in susceptibility to stress. J Neurosci 35:3201-6.

Phillips ML, Robinson HA, Pozzo-Miller L (2019) Ventral hippocampal projections to the medial prefrontal cortex regulate social memory. Elife 8.

Purohit K, Parekh PK, Kern J, Logan RW, Liu Z, Huang Y, McClung CA, Crabbe JC, Ozburn AR (2018) Pharmacogenetic Manipulation of the Nucleus Accumbens Alters Binge-Like Alcohol Drinking in Mice. Alcohol Clin Exp Res 42:879-888.

Roman CW, Derkach VA, Palmiter RD (2016) Genetically and functionally defined NTS to PBN brain circuits mediating anorexia. Nat Commun 7.

Rossetti AC, Serena Paladini M, Colombo M, Gruca P, Lason-Tyburkiewicz M, Tota-Glowczyk K, Papp M, Riva MA, Molteni R (2018) Chronic Stress Exposure Reduces Parvalbumin Expression in the Rat Hippocampus through an Imbalance of Redox Mechanisms: Restorative Effect of the Antipsychotic Lurasidone. Int J Neuropsychopharmacol 21:883-893.

Roth BL (2016) DREADDs for Neuroscientists. Neuron.

Shepard R, Page CE, Coutellier L (2016a) Sensitivity of the prefrontal GABAergic system to chronic stress in male and female mice: Relevance for sex differences in stress-related disorders. Neuroscience 332:1-12.

Shepard R, Page CE, Coutellier L (2016b) Sensitivity of the prefrontal GABAergic system to chronic stress in male and female mice: Relevance for sex differences in stress-related disorders. Neuroscience 332:1-12.

Sokolova I V., Mody I (2008) Silencing-induced metaplasticity in hippocampal cultured neurons. J Neurophysiol 100:690-697.

Soumier A, Sibille E (2014) Opposing Effects of Acute versus Chronic Blockade of Frontal Cortex Somatostatin-Positive Inhibitory Neurons on Behavioral Emotionality in Mice. Neuropsychopharmacology 39:2252-2262.

Stedehouder J, Brizee D, Shpak G, Kushner SA (2018) Activity-dependent myelination of parvalbumin interneurons mediated by axonal morphological plasticity. J Neurosci 38:3631-3642.

Stemmler M, Koch C (1999) How voltage-dependent conductances can adapt to maximize the information encoded by neuronal firing rate. Nat Neurosci 2:521-527.

van Bokhoven H, Selten M, Nadif Kasri N (2018) Inhibitory control of the excitatory/inhibitory balance in psychiatric disorders. F1000Research.

Wiegert JS, Mahn M, Prigge M, Printz Y, Yizhar O (2017) Review Silencing Neurons: Tools, Applications, and Experimental Constraints. Neuron 95:504-529.

Wohleb ES, Terwilliger R, Duman CH, Duman RS (2018) Stress-Induced Neuronal Colony Stimulating Factor 1 Provokes Microglia-Mediated Neuronal Remodeling and Depressive-like Behavior. Biol Psychiatry 83:38-49.

Zhan C, Zhou J, Feng Q, Zhang J en, Lin S, Bao J, Wu P, Luo M (2013) Acute and long-term suppression of feeding behavior by POMC neurons in the brainstem and hypothalamus, respectively. J Neurosci 33:3624-3632.
