# Supplementary figures and images for: Chemogenetic Inhibition of Infralimbic Prefrontal Cortex GABAergic Parvalbumin Interneurons Attenuates the Impact of Chronic Stress in Male Mice
Source: eNeuro. 2020 Oct 26;7(5):ENEURO.0423-19.2020. doi: 10.1523/ENEURO.0423-19.2020 (PMC7598911; doi:10.1523/ENEURO.0423-19.2020)

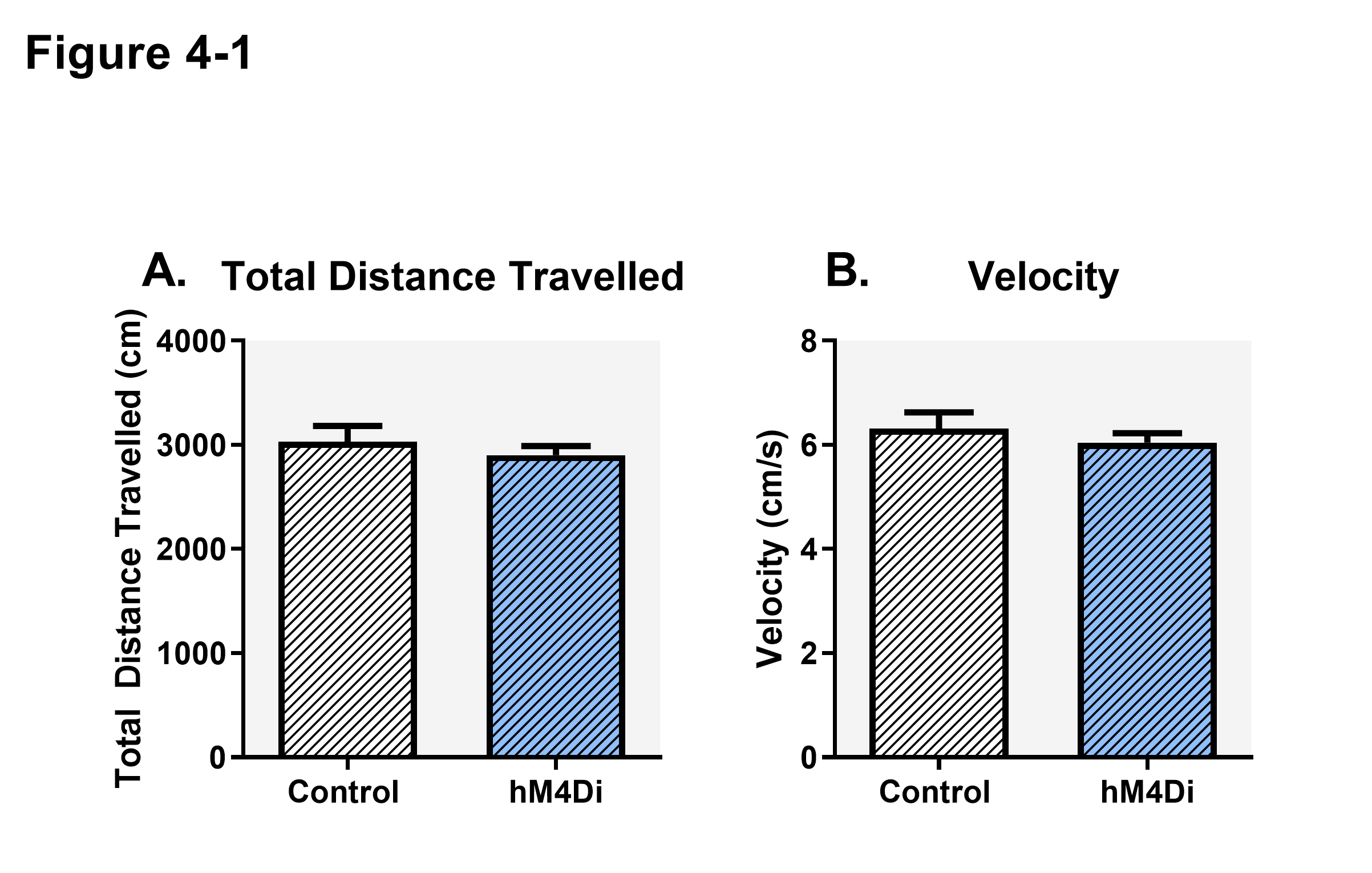

Supplement: Extended Data Figure 4-1 — Effect of chronic inhibition of PV IN on locomotor activity. Chronic inhibition of PV INs had no effect on locomotor activity as demonstrated by no change in distance travelled (A) or velocity (B) in hM4Di group compared with control group in a Y maze task. Values represent mean ± SEM, n = 9–10 per group (p > 0.05). Download Figure 4-1, TIF file. [file enu-eN-NWR-0423-19-s02.tif]

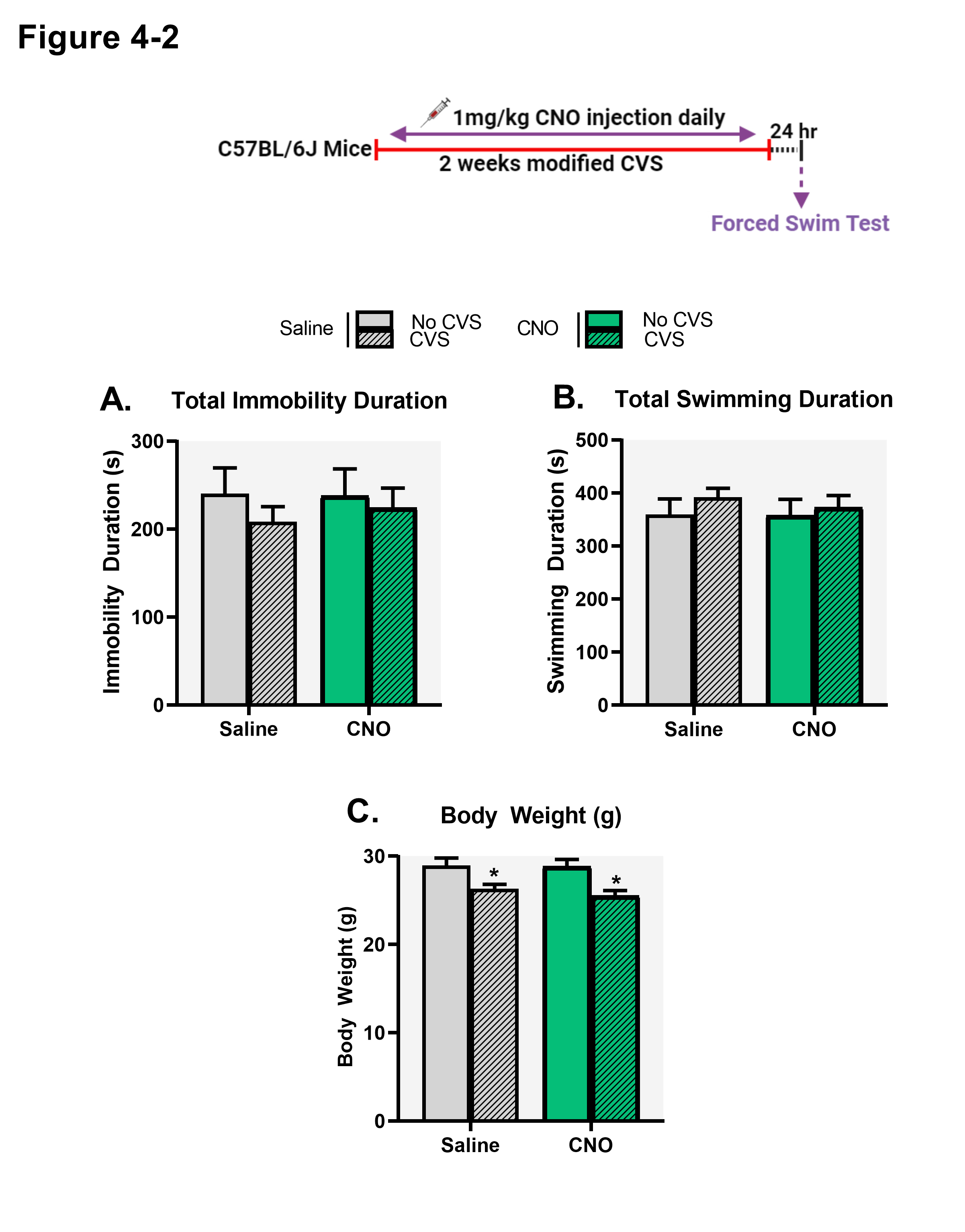

Supplement: Extended Data Figure 4-2 — Effect of chronic dosing of CNO and saline in control and CVS animals in FST. Chronic CNO administration had no effect on immobility (A) or swimming duration (B) in FST and also did not have any effect on body weight (C) in either CVS or Control groups. Values represent mean ± SEM; n = 8 per group (p > 0.05); * indicate planned comparisons significant effect p < 0.05 versus corresponding No CVS Control groups. Download Figure 4-2, TIF file. [file enu-eN-NWR-0423-19-s03.tif]

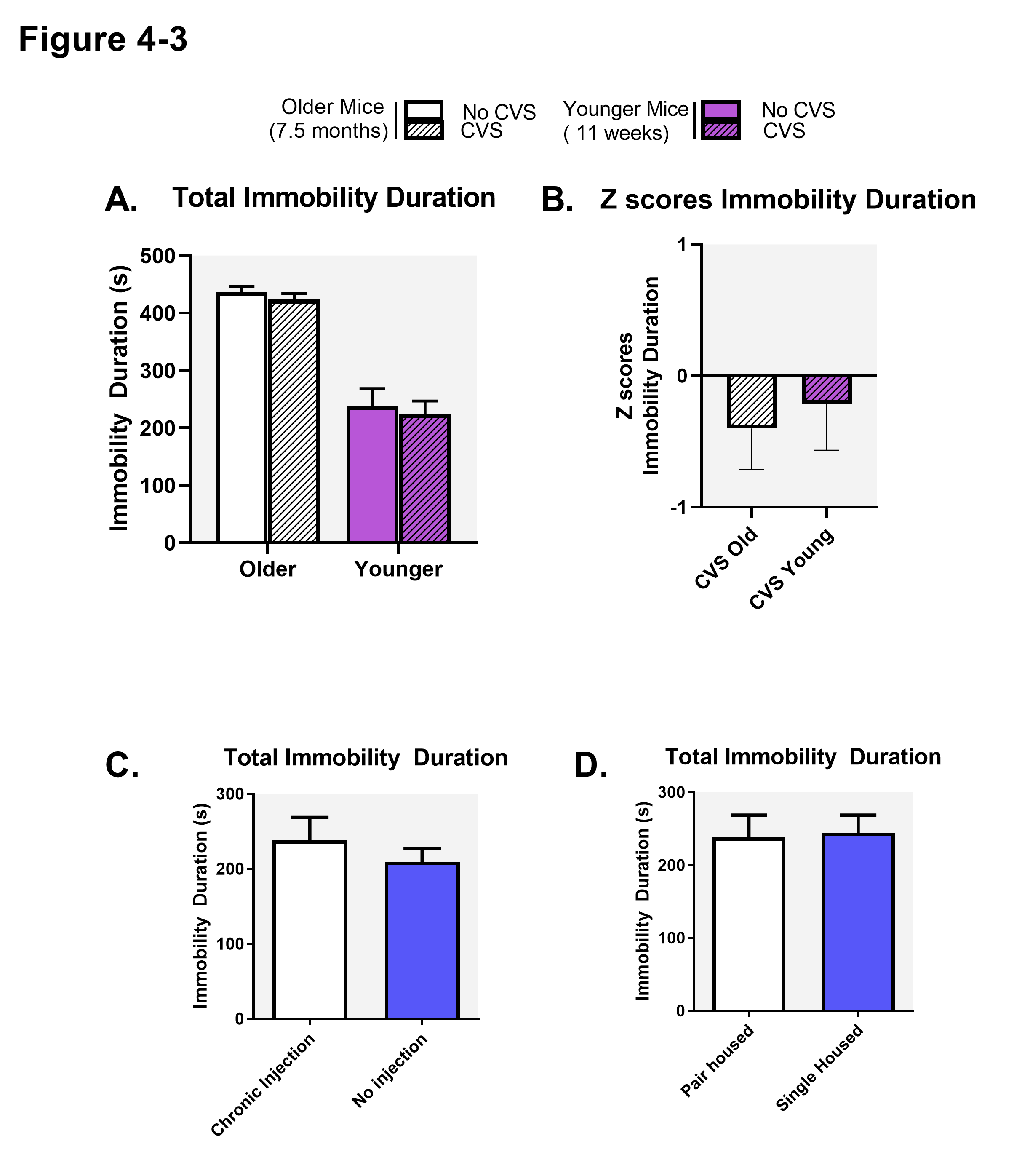

Supplement: Extended Data Figure 4-3 — Effect of age, chronic injection, and housing condition in FST. A, B, Effect of age on FST immobility duration. Analysis of z scores showed that CVS had no effect on FST behavior in different age groups (t = 0.4, df = 16, p = 0.7; B). Younger and older animals were run in separate experiments. Z score calculation was done as described previously using formula z = (X-μ)/σ to indicate how many SDs (σ) each CVS immobility duration value (X) was from the mean of control group (μ) for each age (Guilloux et al., 2011). C, D, Effect of chronic injection stress and housing conditions on immobility duration in FST in control animals. There was no significant effect of chronic injection stress (t = 0.8, df = 14, p = 0.4; C) or housing conditions (t = 0.2, df = 14, p = 0.9; D) in FST behavior. Values represent mean ± SEM; n = 8–10 per group; * indicates significant effect p < 0.05 versus corresponding control group. Download Figure 4-3, TIF file. [file enu-eN-NWR-0423-19-s04.tif]

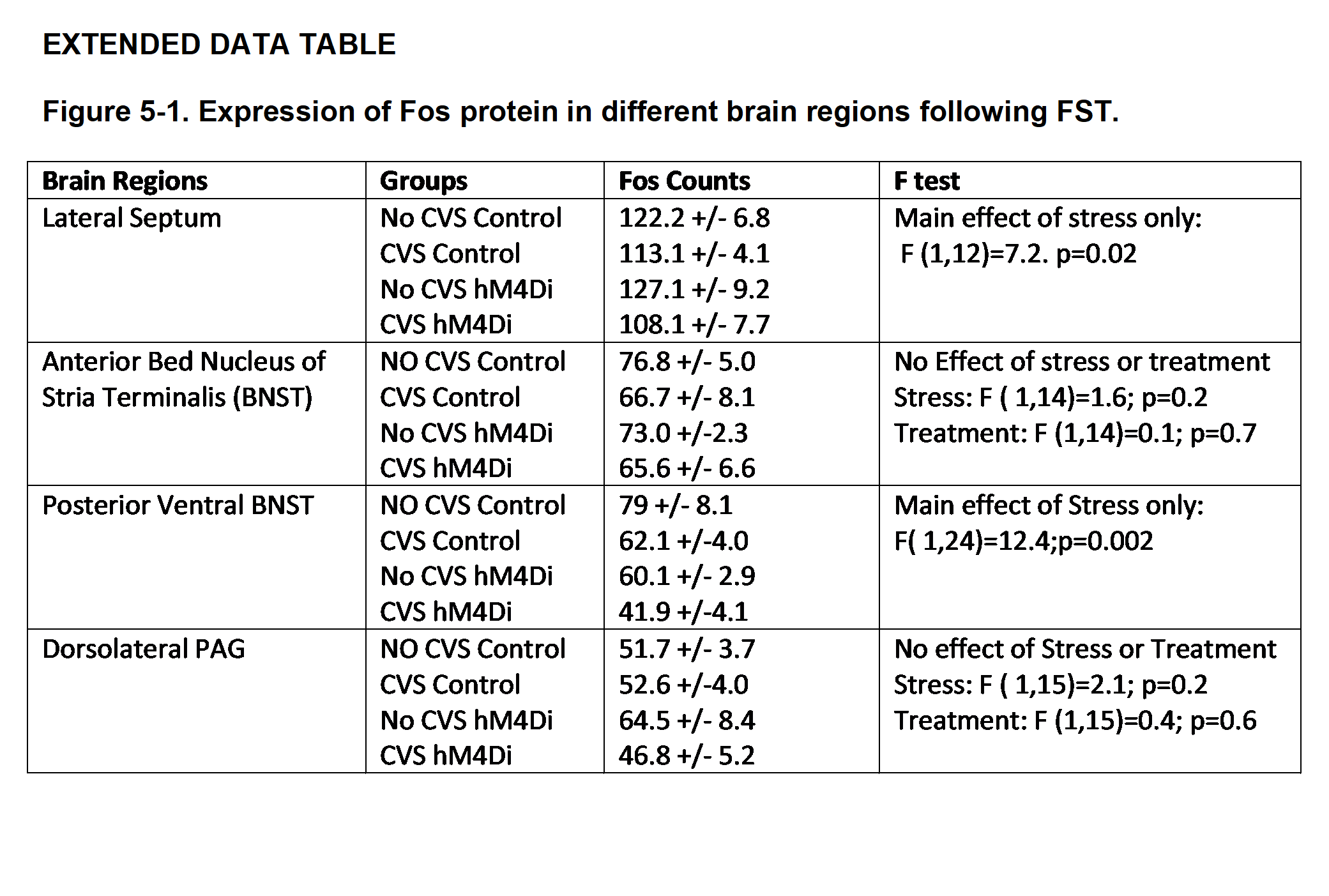

Supplement: Extended Data Figure 5-1 — Fos protein expression in several brain regions following CVS. Figure depicts Fos protein expression in lateral septum, anterior and posterior ventral BNST, and dlPAG. No significant treatment effects of PV IN inhibition was observed in any of the above-mentioned brain regions. Values represent mean ± SEM, n = 7–10 per group. Download Figure 5-1, TIF file. [file enu-eN-NWR-0423-19-s05.tif]
